# Supplementary material for: Prolonged intracellular accumulation of light-inducible nanoparticles in leukemia cells allows their remote activation
Source: Nat Commun. 2017 May 11;8:15204. doi: 10.1038/ncomms15204 (PMC5437273; doi:10.1038/ncomms15204)
Supplement: Supplementary Information — Supplementary Figures, Supplementary Methods and Supplementary References [file ncomms15204-s1.pdf]

Supplementary Figure 1

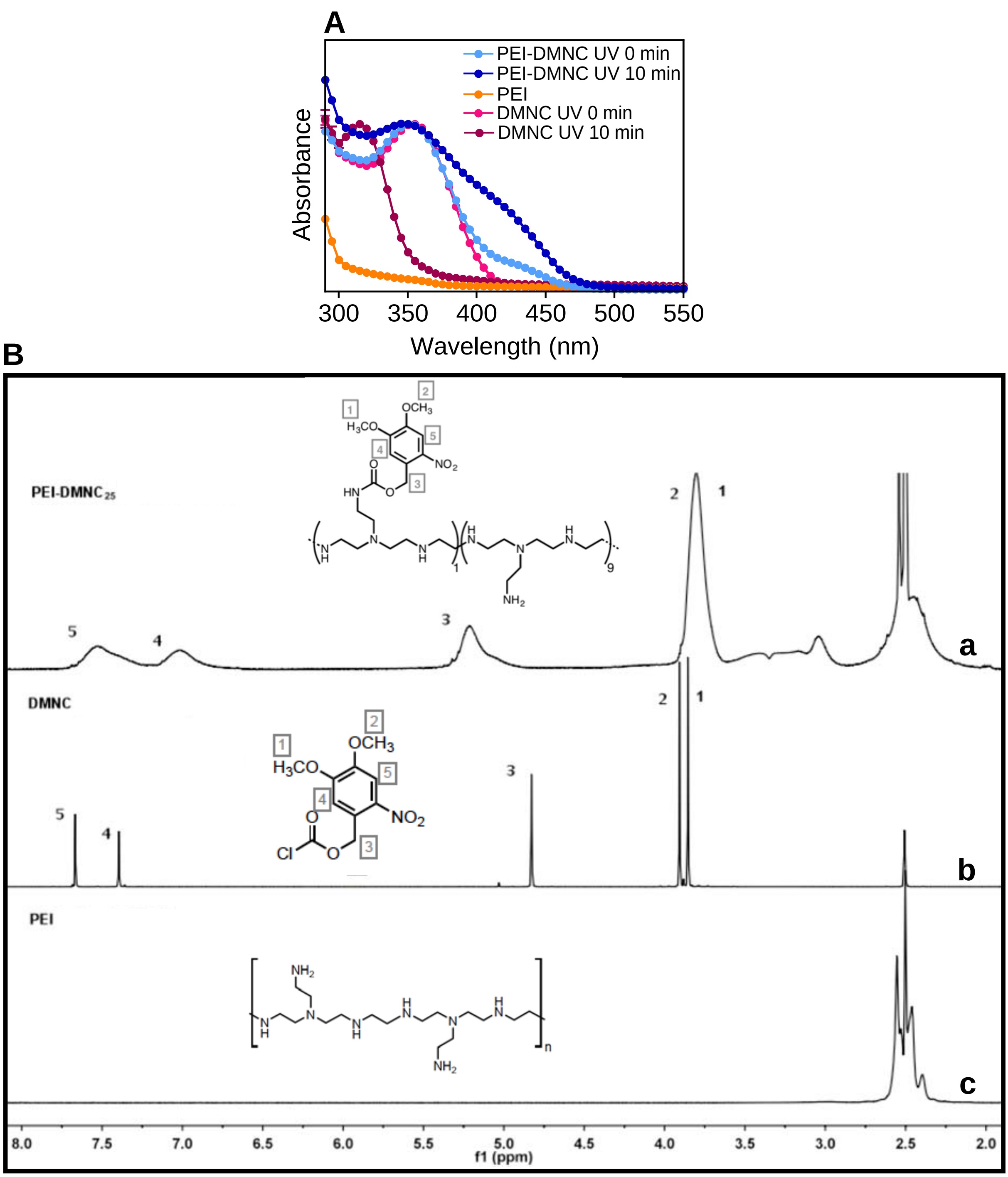

Supplementary Figure 1 – Characterisation of PEI derivatized with DMNC. (A) Effect of UV light (365 nm, 100 Watts) in the absorbance properties of PEI-DMNC (1 mg/mL in DMSO), PEI (1 mg/mL in DMSO) and DMNC (250 µg/mL, in DMSO). The experimental DS of PEI-DMNC was 10% as determined by spectrophotometry, using a calibration curve of DMNC, and by 1H NMR. For DMNC, the absorption maximum at 355 nm reverted to baseline levels after 10 min of UV exposure, indicating the photo-cleavage of DMNC, and a new absorption peak was observed at 320 nm, due to the formation of 4,5-dimethoxy-2-nitrobenzyl alcohol. For PEI-DMNC, there was a decrease in the intensity of the peak at 355 nm and a concomitant increase in the peak at 320 nm; however our results suggest that not all the attached DMNC molecules were photo-cleaved. **(B)** 1H NMR spectra of PEI, DMNC and PEI-DMNC. 1H NMR spectra (in DMSO-d6) of (a) PEI-DMNC conjugate, (b) DMNC and (c) PEI.

Supplementary Figure 2

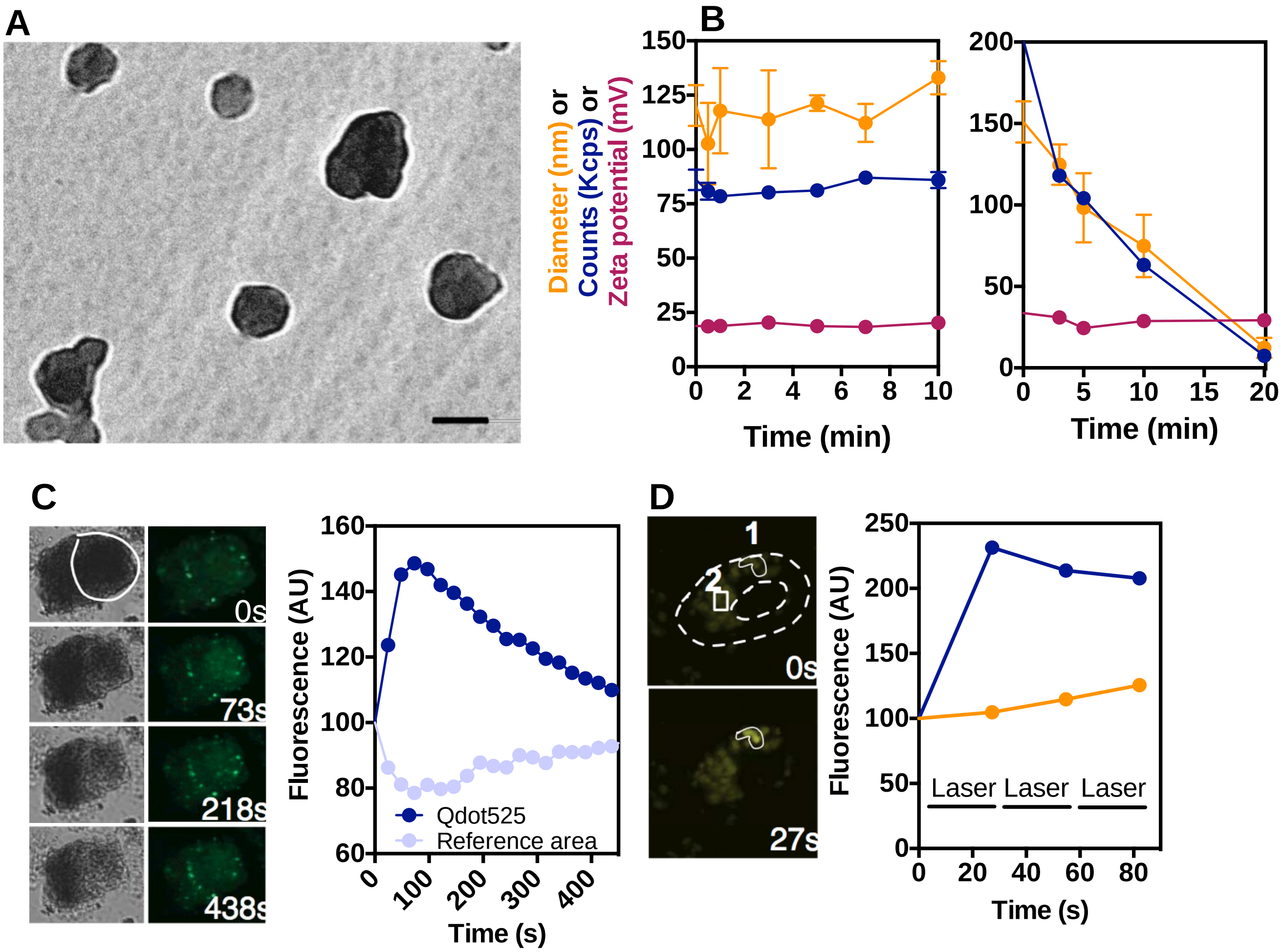

Supplementary Figure 2- Disassembly and release properties of light-activatable NPs. (A) TEM of the light-activatable NPs. Bar represents 100 nm. **(B)** Blue laser (405 nm, 80 mW) activation of NPs with (**right)** and without (**left**) DMNC photochrome. A suspension of NPs (100 µL, 100 µg, in water) was exposed to a blue laser up to 20 min. Then, the NP suspension was diluted up to 50 µg/mL in water and the size, zeta potential and number of NPs (Kcps) in the suspension was evaluated by dynamic light scattering. In **B** results are expressed as Mean ± SEM (n = 5). **(C)** Confocal images showing light-disassembly of quantum dots 525 (Qdot525)-labeled NPs. A section of a NP aggregate (area delimited in the figure) was bleached continuously by a laser at 405 nm as confocal images were collected every 20 s. The images show the disassembly of the bleached area of the NP aggregate. Fluorescence intensity of the area bleached by the laser and reference area (i.e., not activated by the laser) overtime. Our results show that fluorescence intensity increases after light exposure due to the disassembly of the NP and the decrease in the quenching of Qdot fluorescence after NP disassembly. **(D)** Confocal imaging of HUVECs after exposure for 4 h to QDot525-labelled NPs. A small section of the cell (region 1, created by a mask) was then exposed to blue light laser cycles (405 nm) in a Zeiss confocal microscope and the intensity of fluorescence at 525 nm monitored. In parallel, the fluorescence of another section of the cell (region 2) not excited with the laser was monitored as a control. Our results show that the fluorescence intensity in region 2 maintains overtime while in region 1 the intensity increases. Blue dots and line presents the blue light laser-exposed area of Qdot525-labelled NPs; orange dots and line presents the control unexposed area of Qdot525-labelled NPs. Dashed areas show cell membrane and nucleus.

Supplementary Figure 3

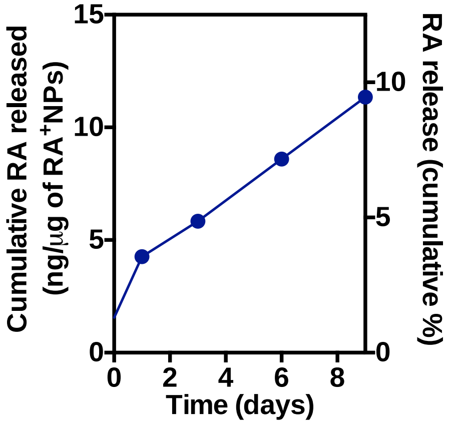

Supplementary Figure 3- Release of RA from light-activatable RA+NPs without light activation. Nanoparticles (2.5 mg) were placed in PBS (0.5 mL) and incubated under mild agitation at 37 °C. At specific intervals of time, the nanoparticle suspension was centrifuged (at 14.000 g for 3 min) and 0.4 mL of the release medium removed and replaced by a new one. The reserved supernatant was stored at 4°C until the RA content in release samples was assessed by spectrophotometry at 350 nm. Concentrations of RA were determined by comparison to a standard curve. All analyses were conducted in triplicate. SEM is smaller than the symbols.

Supplementary Figure 4

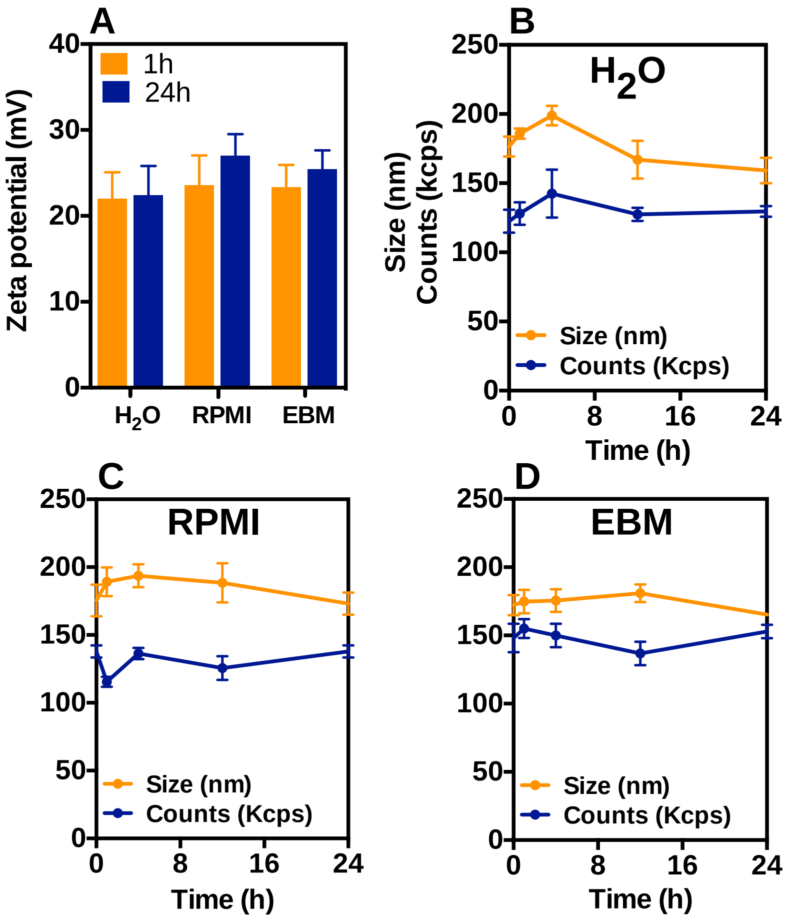

Supplementary Figure 4- Stability of RA+NPs suspended in water or cell culture media. (A) Zeta potential of RA+NPs suspended in water, serum-free RPMI (medium used for leukemia cells) or EBM medium (medium used for HUVECs). It should be noted that the transfection of the cells with RA+NPs was always performed in serum-free media. Diameter (nm) and counts (Kcps) of RA+NPs suspended in water **(B)**, RPMI medium **(C)** or EBM medium **(D)**. A suspension of RA+NPs (2 mL, 25 µg/mL) was prepared and diameter, counts and zeta potential determined by dynamic light scattering method (DLS) using a Zeta Plus Analyzer (Brookhaven). Results are expressed as Mean ± SEM (*n* = 3).

Supplementary Figure 5

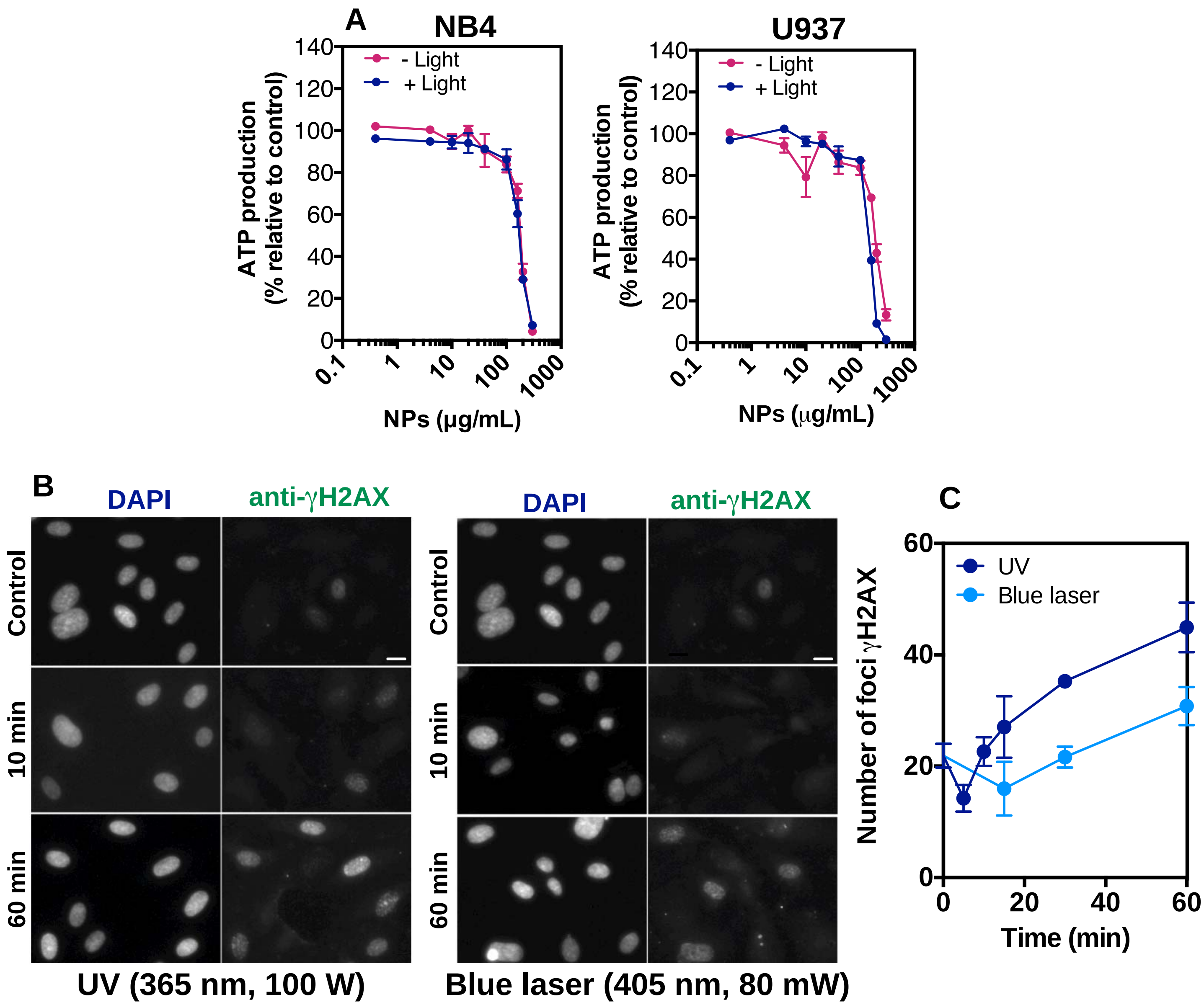

Supplementary Figure 5- Cytotoxicity of RA+NPs and light. (A) Cytotoxicity of RA+NPs against NB4 and U937 cells. Cells were cultured in medium supplemented with light-sensitive RA+NPs for 4 h, washed, exposed or not to a UV light for 10 min, and then cultured for 20 h. Cell cytotoxicity was evaluated by an ATP kit. Results are expressed as Mean ± SEM (n = 3). **(B)** HUVECs were exposure to 10 min or 60 min of UV light (365 nm, 100 W) (left panel) or blue light (405 nm, 80 mW) (right panel) and allowed to recover for 6 h. Cells were then fixed and stained to identify γH2AX-containing foci, as biomarker for nuclear sites of DNA damage in affected cells. Bar corresponds to 10 µm. **(C)** Time-dependent increase of γH2AX after UV light (365 nm, 100 W) or blue light (405 nm, 80 mW) irradiation. Quantitative analysis of foci intensity were quantified using imageJ software and normalised to the control condition. Results are expressed as Mean ± SEM. The calculations were performed in 5 different images for a total of ca. 250 cells (ca. 50 cells per image).

Supplementary Figure 6

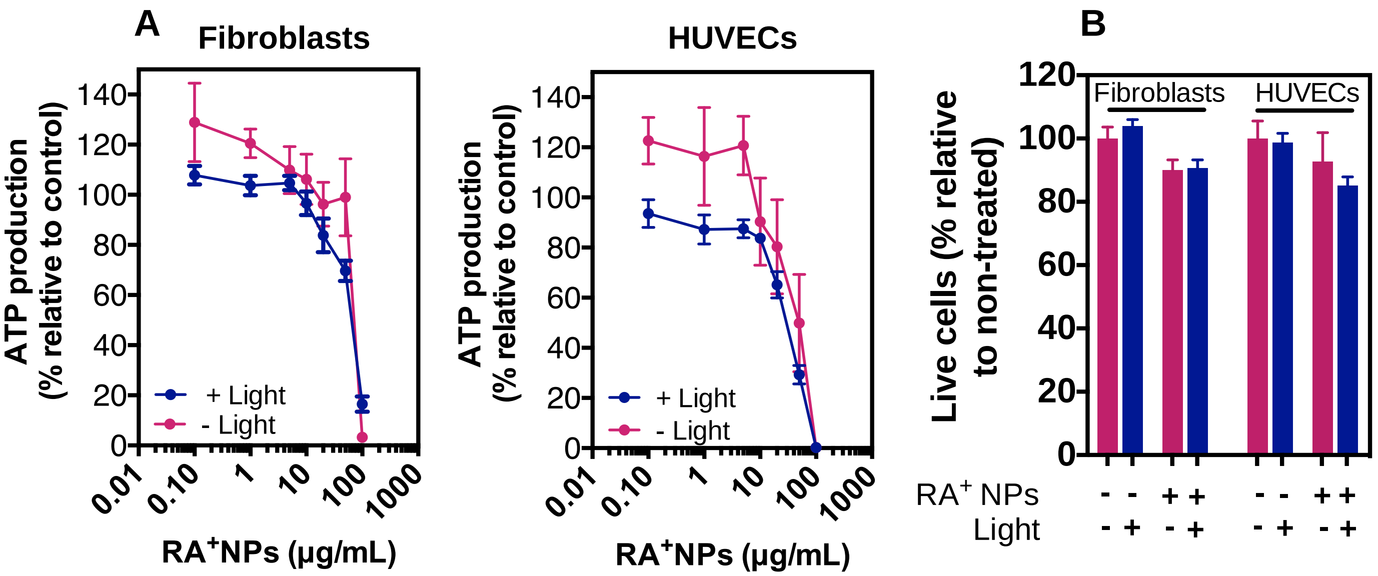

Supplementary Figure 6- Cytotoxicity of RA+NPs in non-leukemic cells. Cytotoxicity of RA +NPs against human vein umbilical endothelial cells (HUVECs) or human dermal fibroblasts. Cells were cultured in medium supplemented with light-sensitive RA+NPs for 4 h, washed, exposed or not to a UV light for 5 min, and then cultured for 20 h. Cell cytotoxicity was evaluated by an ATP kit **(A)** or Annexin V/PI staining **(B)** followed by flow cytometry analyses. In this case, the concentration of RA+NPs was 10 µg/mL. Live cells were negative for Annexin V and PI staining. Results are expressed as Mean ± SEM (n = 3).

Supplementary Figure 7

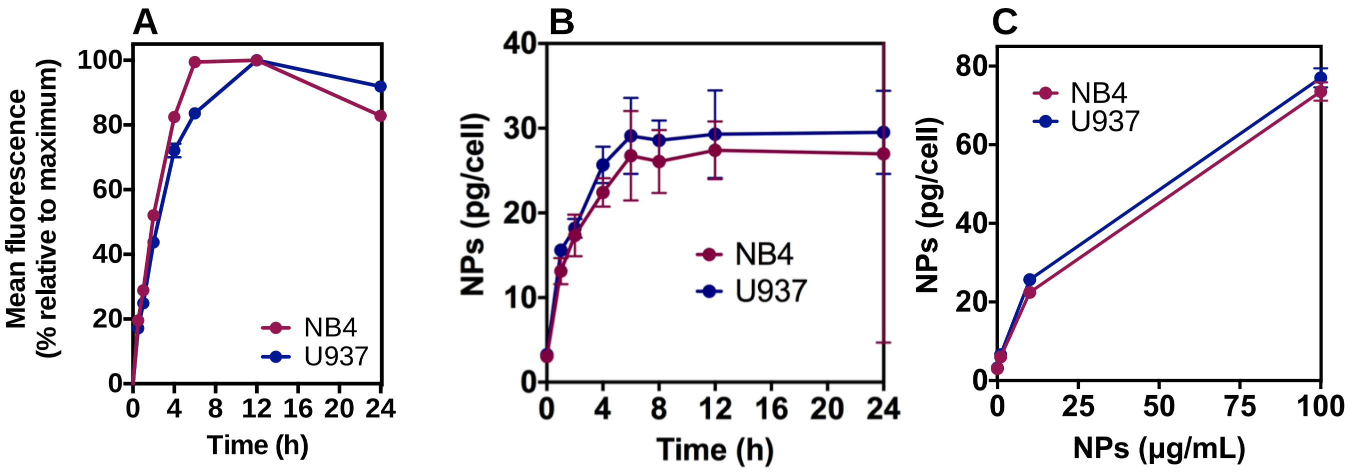

Supplementary Figure 7- Cellular uptake of RA+NPs. (A) Uptake of TRITC-labeled RA +NPs (10 µg/mL) in leukemia cells as determined by FACS. Cells were cultured in medium supplemented with NPs for the time defined in the graph, washed and characterised by flow cytometry. The results are expressed as Mean ± SEM (n = 3). **(B-C)** Quantification of RA+NP internalization in leukemia cell lines NB4 and U937 as determined by ICP-MS analysis (Zn quantification). Cells were incubated with 10 µg/mL NPs (B and C) or 100 µg/mL NPs (C) up to 24 h. After each incubation period, the cells were extensively washed with PBS followed by the addition of an aqueous solution of nitric acid (1 mL, 69% (v/v)). The concentration of intracellular levels of Zn was quantified by ICP-MS. The concentration was normalised per cell. The estimation of NPs was done based on standard solutions. The results are expressed as Mean ± SEM (n=3).

Supplementary Figure 8

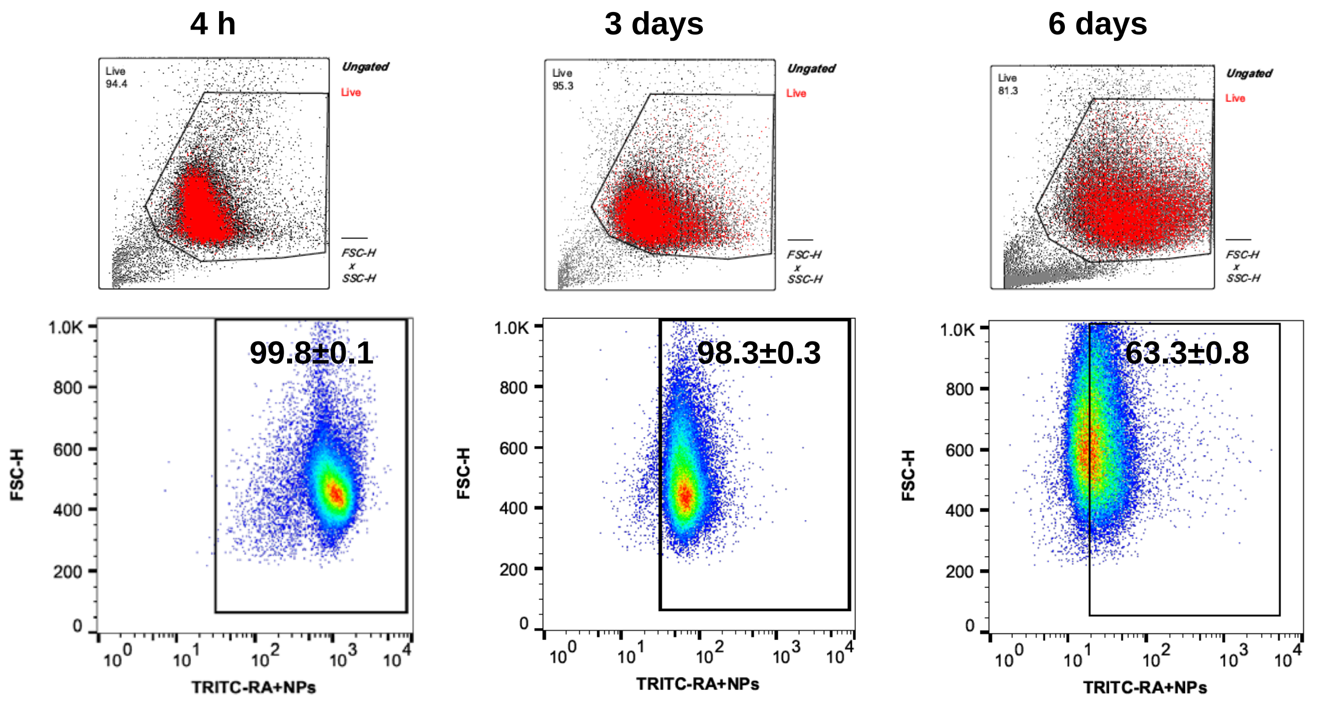

Supplementary Figure 8- Dilution of RA+NPs during NB4 cell culture. RA+NP dilution with cell culture was monitored over 6 days by flow cytometry. NB4 cells (500.000 cells/mL) were plated in 6 well plates and incubated in serum-free RPMI-1640 with RA+NPs (20 µg/ mL). After 4 h incubation, cells were washed three times with PBS to remove RA+NPs not internalised and the cells were left to grow at 200.000 cells/mL in complete medium for additional 4 h, 3 days and 6 days. After each incubation, cells were counted, collected by centrifugation and ressuspended in PBS for flow cytometry evaluation.

Supplementary Figure 9

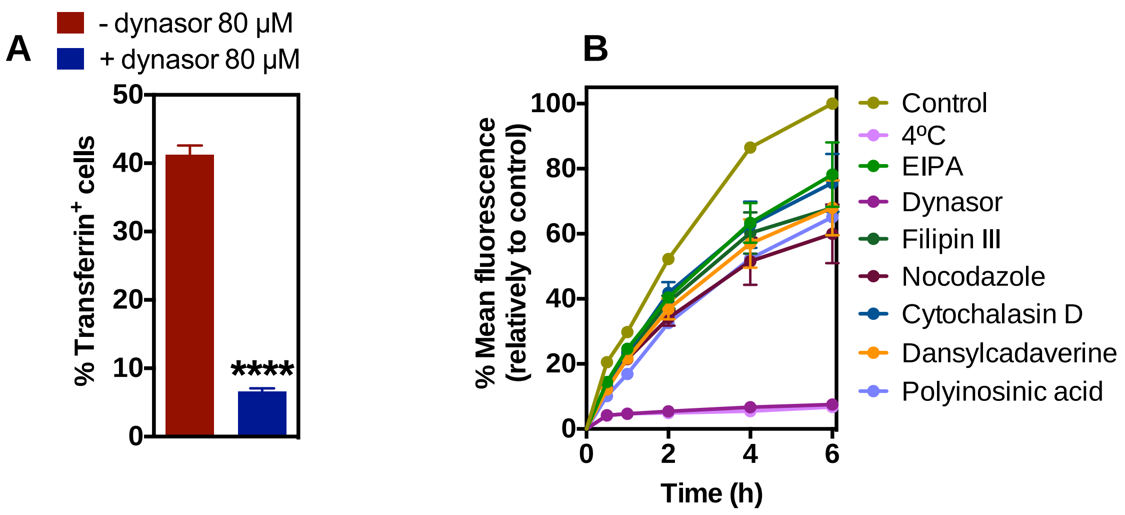

Supplementary Figure 9- Internalization mechanism of RA+NPs in Zn-induced U937 cells. (**A)** Transport of FITC-labeled transferrin (1 µg/mL) known to selectively enter cells via clathrin-mediated endocytosis. Dynasor at concentration of 80 µM inhibits the internalisation of transferrin in U937 cells. Cells were exposed to culture medium with and without dynasor for 30 min, exposed to FITC-labeled transferrin for 3 min, at 4 ºC, and finally characterized by FACS. Results are expressed as Mean ± SEM (n = 3). Statistical analyses were performed by an unpaired t-test. **** Denotes statistical significance ($P<0.0001$). **(B)** Uptake of TRITC-labeled RA+NPs by U937 cells in the presence of several endocytosis inhibitors. Filipin III inhibits cholesterol dependent internalization mechanisms, ethylisopropylamiloride (EIPA) inhibits macropinocytosis, nocodazole inhibits microtubule dependent pathways, cytochalasin D inhibits all pathways dependent on actin (including macropinocytosis), dansylcadaverine and dynasore inhibits clathrin-mediated endocytosis and polyinosinic acid inhibits scavenger receptors (Mukherjee, S., et al., Physiol Rev 1997, 77, 759-803). Results are expressed as Mean ± SEM (n = 3).

Supplementary Figure 10

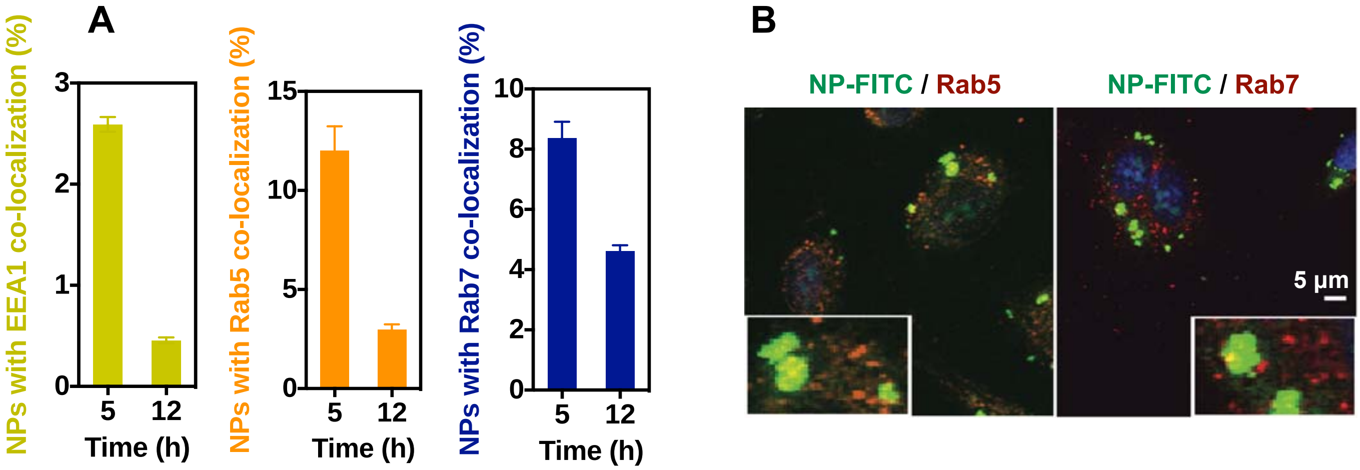

Supplementary Figure 10- Intracellular trafficking of FITC-labeled RA+NPs. HUVECs were incubated with FITC-labeled RA+NPs (1 µg/mL) for 4 h, washed extensively, cultured in EGM-2 medium for 1 or 8 additional hours and fixed. Early endosome were stained with EEA1 antibody, early/late endosomes were stained with Rab5 antibody and late endosome/lysosomes were stained with Rab7 antibody. **(A)** Quantification of FITC-labelled RA+NPs co-localized with EEA1, Rab5 and Rab7. Results are expressed as Mean ± SEM (n = 70-100 cells). At 5 h, there is a clear accumulation of FITC-labeled NPs within vesicles that are mostly Rab5 and/or Rab7 positive with very low EEA1 co-localization. The high co-localization with Rab5 and the size of the vesicles containing NPs (see B) suggests that macropinocytosis is also an entrance route for these NPs. **(B)** Representative images of the intracellular distribution of FITC-labelled RA +NPs at time 5 h in relation to early/late endosomes stained with Rab5 antibodies (left image), and late endosome/lysosome stained with Rab7 antibody (right image).

Supplementary Figure 11

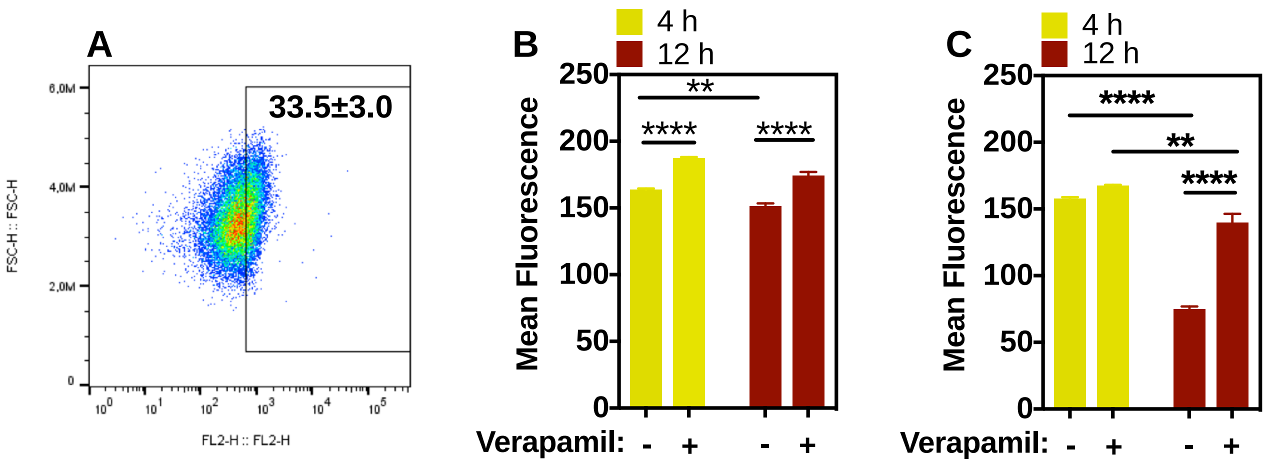

Supplementary Figure 11- Effect of inhibition of P-gp in the accumulation of NPs. (A) Expression of P-gp in Zn-induced U937 cells as evaluated by flow cytometry. Percentage of positive cells was calculated based in the isotype control. (**B**) TRITC-labeled RA+NPs (10 µg/mL) or (**C**) TRITC-labeled USPIO NPs (100 µg/mL) intracellular accumulation in Zn-induced U937 cells in the presence of the P-gp antagonist verapamil. Cells were exposed to culture medium with verapamil, TRITC-labeled NPs for 4 h, cultured for additional 8 h and finally characterized by flow cytometry. In A, B and C, results are expressed as Mean ± SEM, n=3. Statistical analyses were performed using a One-Way Anova followed by a Newman-Keuls post-test.*, **, ***, **** denotes statistical significance (P<0.05, P<0.01, P<0.001, P<0.0001).

Supplementary Figure 12

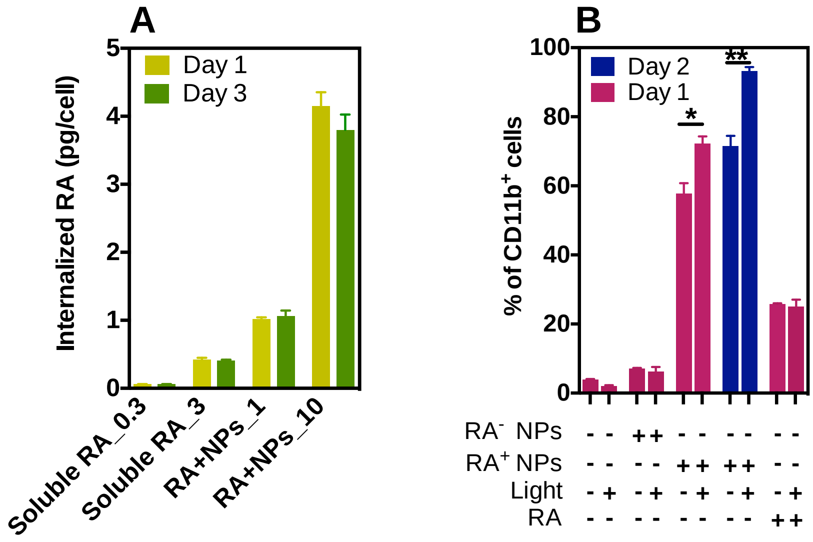

Supplementary Figure 12- (A) Uptake of RA by U937 cells. U937 cells were cultured with soluble [3H]-RA (0.3 or 3 µg/mL) in culture medium for the entire duration of the experiment, or light-activatable [3H]-RA+NPs (1 and 10 µg/mL). NPs were added to cell culture for 4 h. Then, the cells were washed with PBS, and fresh cell medium added and the cells remained in culture for 24/72 h before scintillation counting. **(B)** Myelocytic differentiation of human THP-1 cells. THP-1 cells were cultured with soluble RA (3 µg of RA per mL) in culture medium for the entire duration of the experiment, or light-activatable RA+NPs (10 µg of NPs/mL, i.e., 1.2 µg of RA per mL), or light-activatable NPs without RA (10 µg of NPs/mL). In the case of cells treated with NPs, cells were treated with RA+NPs for 4 h, washed, activated with UV light (365 nm, 100 Watts) for 5 min, and then cultured up to 24 h or 48 h. In case of cells treated with soluble RA, cells were cultured in media containing soluble RA for the entire period of culture. Results are expressed as Mean ± SEM (n = 3). Statistical analyses were performed by an unpaired t-test. *, ** denotes statistical significance (*P*<0.05, *P*<0.01).

Supplementary Figure 13

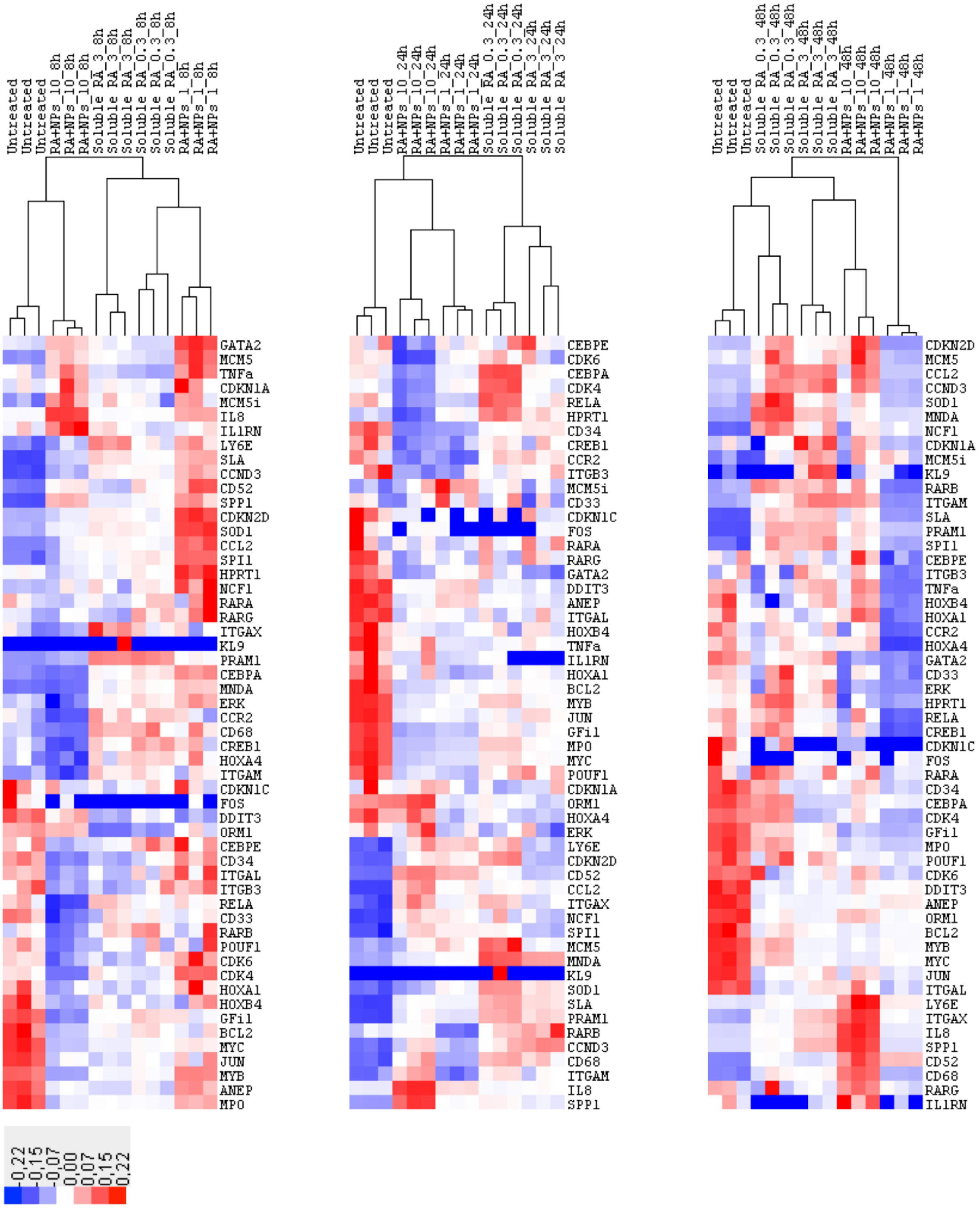

Supplementary Figure 13- Heat map of the differentiation pattern of NB4 cells after exposure to soluble RA (0.3 or 3 µg/mL) or light-activatable RA+NPs (1 or 10 µg/mL). Cells were treated with RA+NPs for 4 h, washed, activated with UV light (365 nm, 100 W) for 5 min, and then cultured for up to 48 h. In case of cells treated with soluble RA, cells were cultured in media containing soluble RA for the entire period of culture. Cells were then lysed and gene expression profile monitored by qRT-PCR using a Fluidigm equipment. A red-blue color scale was used to reflect standardized gene expression, with red indicating higher expression and blue indicating lower expression. Surprisingly, our clustering analysis indicate that NB4 treated with RA+NPs for the first 24 h were more related to the non-treated cells than soluble RA. Yet this effect is inverted for 48 h. Therefore, our data suggests that the most significant alterations in NB4 cells after treatment with RA+NPs occurs between 24 and 48 h.

Supplementary Figure 14

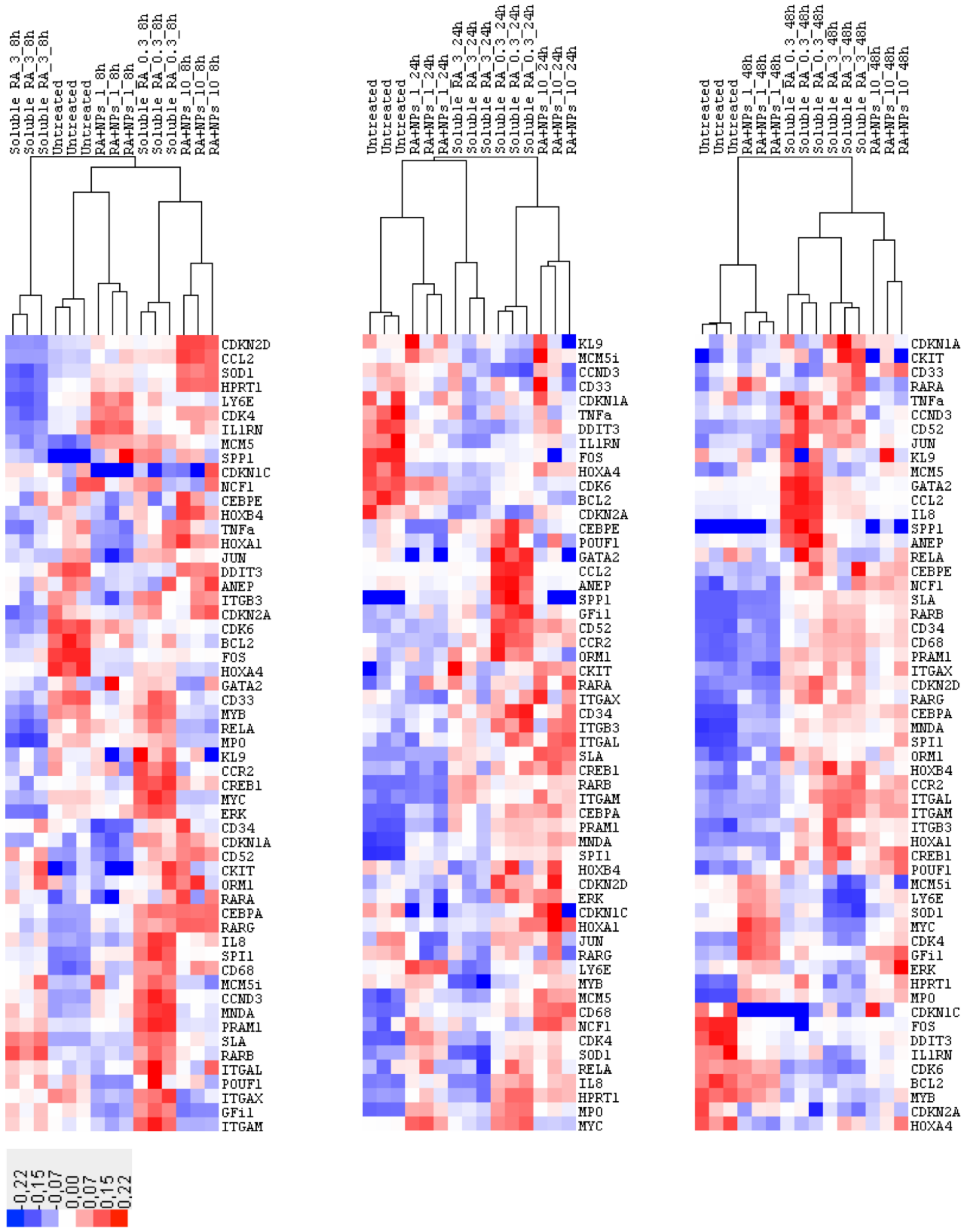

Supplementary Figure 14- Heat map of the differentiation pattern of Zn-induced U937 cells after exposure to soluble RA (0.3 or 3 µg/mL) or light-activatable RA+NPs (1 or 10 µg/mL). Cells were treated with RA+NPs for 4 h, washed, activated with UV light (365 nm, 100 Watts) for 5 min, and then cultured for up to 48 h. In case of cells treated with soluble RA, cells were cultured in media containing soluble RA for the entire period of culture. Cells were then lysed and gene expression profile monitored by qRT-PCR using a Fluidigm equipment. A red-blue color scale was used to reflect standardized gene expression, with red indicating higher expression and blue indicating lower expression. Our clustering analysis indicate that Zn-induced U937 cells treated with 10 µg/mL, but not 1 µg/mL, of RA+NPs were more distant to the non-treated cells than soluble RA. This effect was observed for all the times investigated. Therefore, in contrast to NB4 cells, our clustering analyses indicate that Zn-induced U937 cells were more sensitive to RA released from RA+NPs at least at high concentrations (10 µg/mL).

Supplementary Figure 15

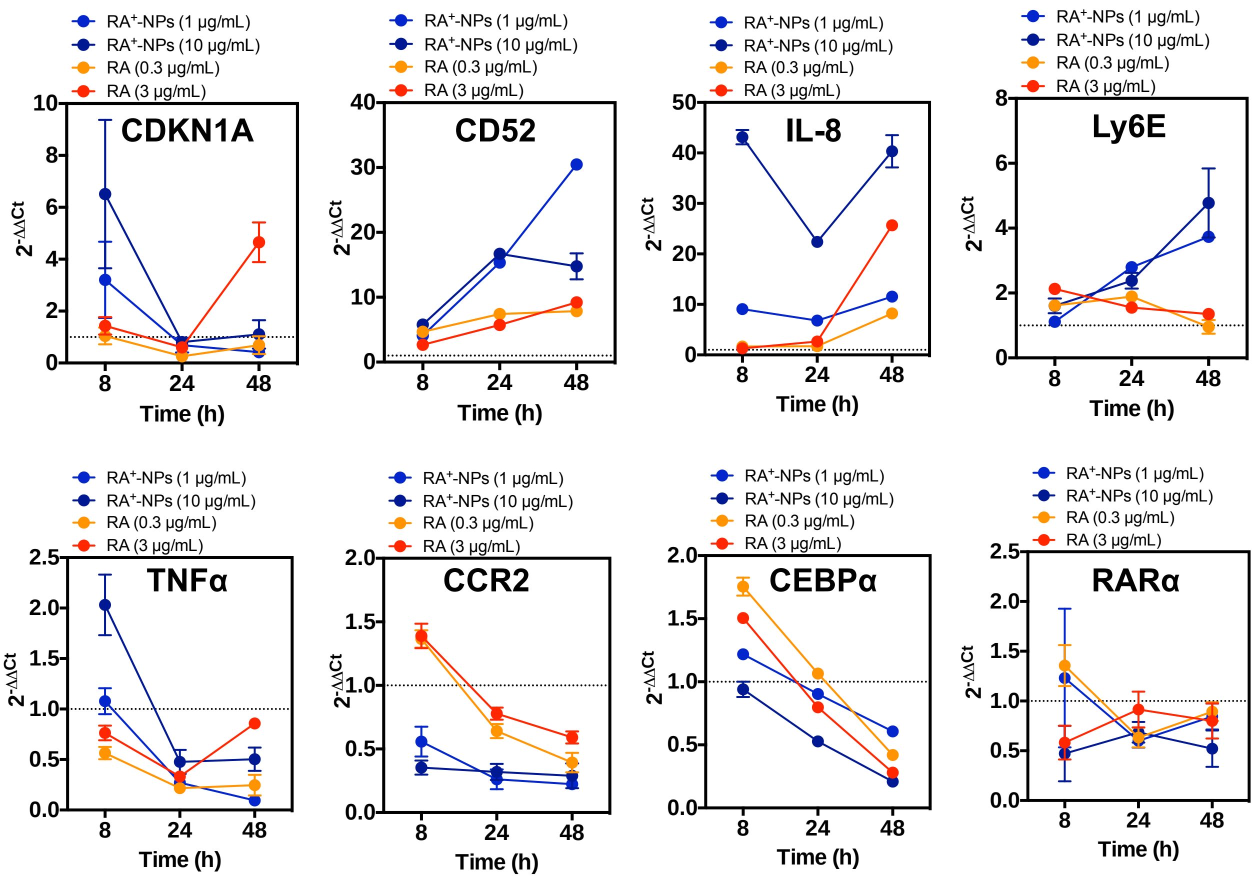

Supplementary Figure 15- Genes modulated by RA during differentiation of NB4 cells as assessed by qRT-PCR analyses. Results are expressed as Mean ± SEM (n=3). In each run, the expression of each gene was normalised by GAPDH gene. Gene expression in each experimental group was normalised by the corresponding gene expression observed in non-treated NB4 cells.

Supplementary Figure 16

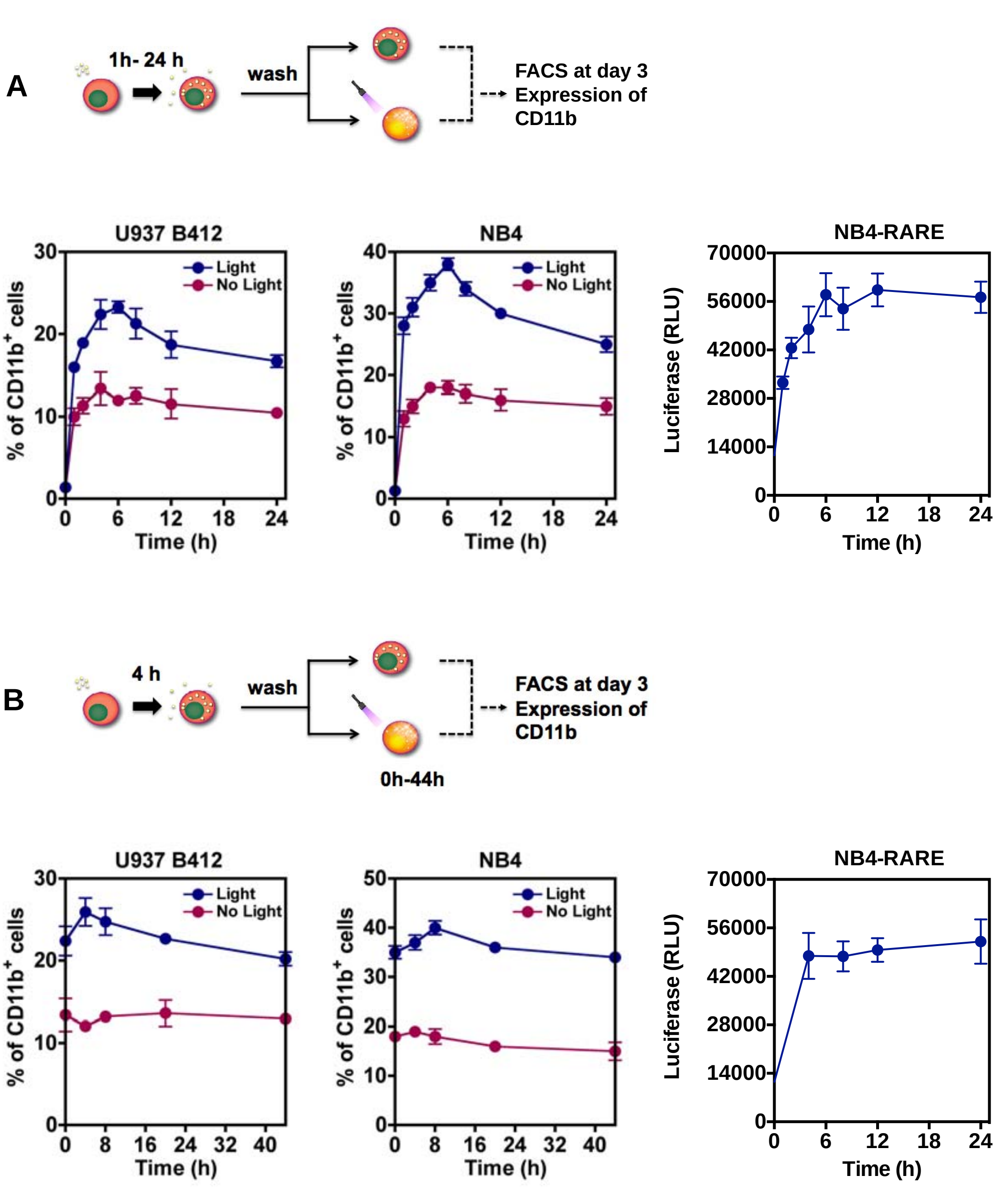

Supplementary Figure 16- Effect of time in the activation of RA+NPs within the cells. (A) Zn-induced U937-B412, NB4 or NB4-RARE-luciferase reporter cells were cultured with RA+NPs (1 µg/mL) for variable period of times (1 up to 24 h), washed with PBS, resuspended in cell culture media, exposed to UV light (365 nm, 100 Watts) for 5 min, and cultured for 12 h (luciferase measurements) or 72 h (flow cytometry analyses). Results are expressed as Mean ± SEM (n = 3). **(B)** Zn-induced U937-B412, NB4 or NB4-RARE-luciferase reporter cells were cultured with RA+NPs (1 µg/mL) for 4 h, washed with PBS, resuspended in cell culture media, exposed to UV light for 5 min at variable periods of time (0 up to 44 h), and cultured for 12 h (luciferase measurements) or 72 h (flow cytometry analyses). Results are expressed as Mean ± SEM (n = 3). In NB4-RARE, the activation of RA-dependent signaling pathway was measured by luminescence while in U937 B412 and NB4, cell differentiation was evaluated by the expression of CD11b by flow cytometry.

Supplementary Figure 17

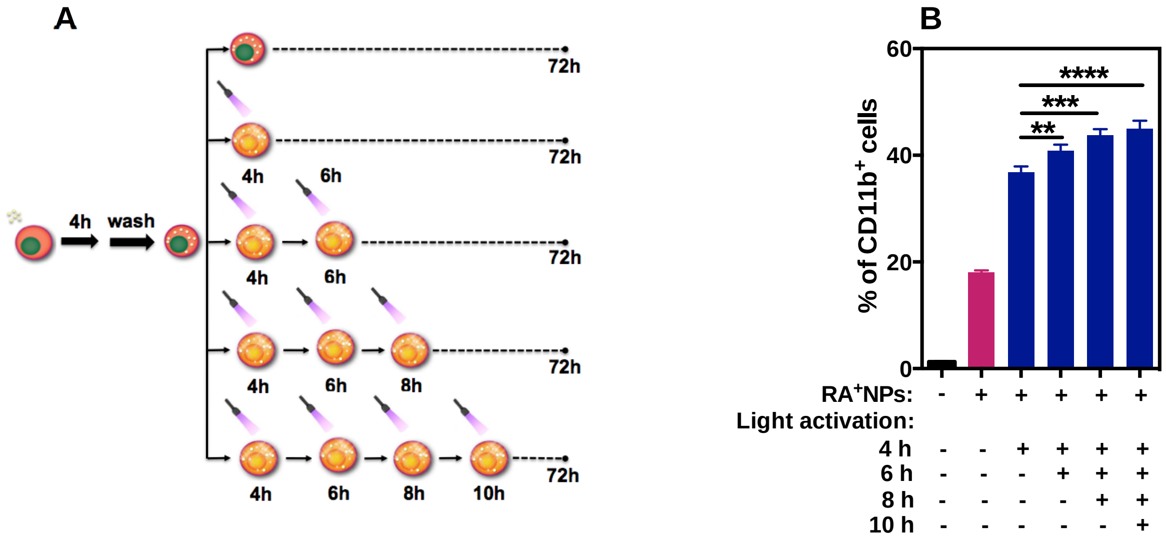

Supplementary Figure 17- Effect of multiple light activation in the differentiation of Zn-induced U937-B412 cells. (A) Schematic representation of the methodology. Cells were cultured with RA+NPs (10 µg/mL) for 4 h, washed with PBS, resuspended in cell culture media, exposed to multiple 5 min-cycles of UV light (365 nm, 100 Watts) during the 72 h of culture. **(B)** Myelocytic differentiation at 72 h, as assessed by the expression of CD11b (by flow cytometry), of human leukemia Zn-induced U923-B412 cells. Results are expressed as Mean ± SEM (n = 3). Statistical analyses were performed using a One-Way Anova followed by a Newman-Keuls post-test. * and ** denotes statistical significance (P<0.05, P<0.01).

Supplementary Figure 18

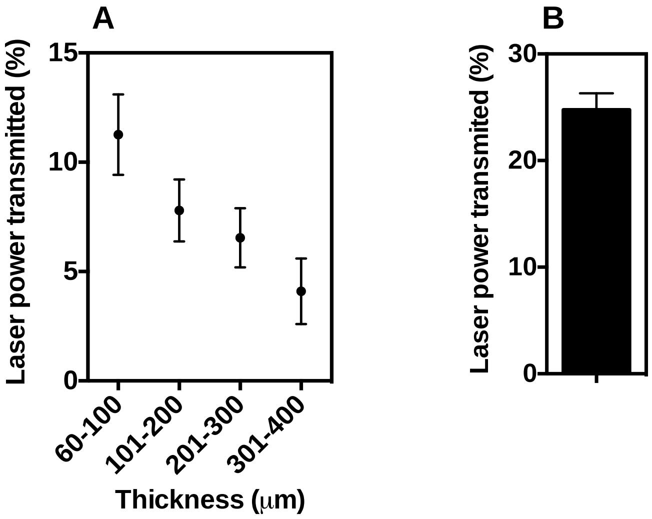

Supplementary Figure 18- Blue laser (405 nm) attenuation through murine skin or calvaria. NOD/SCID mice were euthanised followed by hair shaving and removal of dorsal and ventral skin as well as calvaria bone. Both tissues were kept in cold PBS until further analyses. For the laser attenuation studies, 2 cm x 2 cm skin or 0.5 cm x 0.5 cm calvaria were placed in a plastic petri dish on top of a thermal power sensor (Thorlabs, s310c). Both tissues were then irradiated with a 405 nm laser at 80 mW during 1 min. Laser attenuation values were calculated by normalising against laser power values obtained with the empty petri dish. The thickness of the skin and bone was measured by a caliper. **(A)** Blue laser attenuation as a function of skin thickness. Results are expressed as Mean ± SEM (*n* = 2-6 per each thickness interval). **(B)** Blue laser attenuation in calvaria bone (≈ 260 μm). Laser attenuation in calvaria bone is lower than the skin likely due to the high transparency of the bone. Results are expressed as Mean ± SEM (*n* = 4).

Supplementary Figure 19

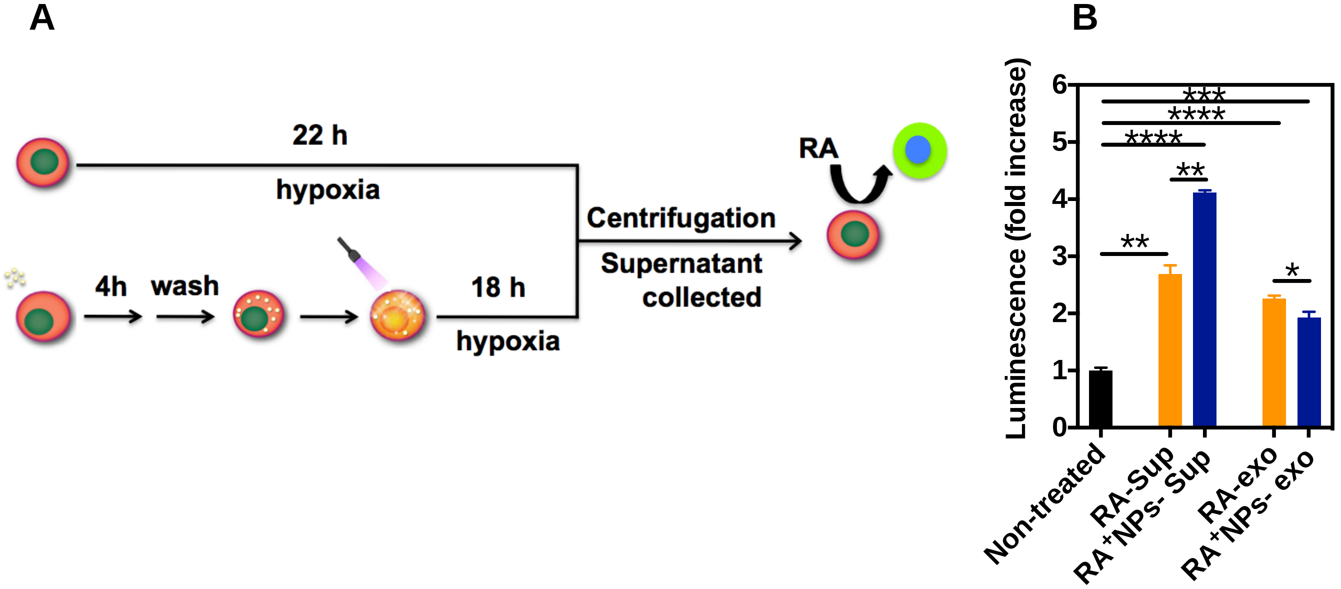

Supplementary Figure 19- Paracrine effect of cells treated with RA+NPs. (A) Schematic representation of the assay. NB4 cells (3,000,000/mL) were grown in RPMI supplemented with 10% (v/v) FBS, penicillin and streptomycin. Cells were treated with RA+NPs (10 µg/mL) for 4 h, washed with PBS to remove the NPs that were not internalized, activated by a blue laser for 5 min, and then cultured for 18 h under hypoxia conditions (0.5% O2) in exosome-depleted medium. A separate group of cells was treated with RA (3 µg/mL) for 22 h in hypoxia conditions (0.5% O2) in exosome-depleted medium. Then, in both groups, cells were centrifuged at 10.000 g (4ºC; 30 min) and part of the supernatant (named in the plot as "Sup") added to NB4 RARE cells (100,000/mL; 1 mL of the Sup). Alternatively, in both groups, cells were centrifuged in multiple steps (please see Supplementary Information) to remove exosomes (named in the plot as "exo") and added to NB4 RARE cells (100,000/mL; 5 µg/mL de exosomes per mL of media) plated in a 24 well plate. After 24 h, the luciferase assay was performed according to instructions of the luciferase kit (britelite™). **(B)** Activation of NB4 RARE reporter cell line with the factors secreted by the NB4 cells for 22 h cultured with soluble RA or RA+NPs. Results are expressed as Mean ± SEM (*n* = 3). Statistical analyses were performed using a One-Way Anova followed by a Newman-Keuls post-test. *, **, *** and **** denotes statistical significance (*P*<0.05, *P*<0.01, *P*<0.001, *P*<0.0001, respectively).

SUPPLEMENTARY METHODS

Preparation and characterization of poly(ethyleneimine) (PEI) conjugated with 4,5-dimethoxy-2-nitrobenzyl chloroformate (DMNC). DMNC (48.5 mg, Sigma) was slowly added to a solution of PEI in DMSO (2 mL containing 50 mg/mL PEI, Sigma) containing triethylamine (24.5 μL, Sigma), and the reaction flask cooled to 0 ºC by immersion on ice. Then, the reaction was allowed to proceed for 24 h at 25 ºC with stirring. At the end, the PEI-DMNC conjugate was purified by dialysis (Spectra/Por® 1 Regenerated Cellulose dialysis membrane, MWCO 6000-8000 Da, Spectrum) against DMSO overnight at room temperature. Reaction yields above 54 % were obtained. For NMR characterization, PEI-DMNC (in DMSO) was precipitated in water, washed, freeze-dried, and then dissolved (10 mg/mL) in DMSO-d6 and ^{1}H NMR spectra were acquired using a Bruker Avance III 400 MHz spectrometer.

Preparation of NPs. Non-activatable NPs were prepared by the electrostatic interaction of PEI (polycation) with dextran sulfate (DS, polyanion) in water, at room temperature, as previously described by us[1]. Briefly, an aqueous DS solution (1 mL, 10 mg/mL) was added drop-by-drop to an aqueous solution of PEI (5 mL, 10 mg/mL) and stirred for 5 min. Then, an aqueous solution of $ZnSO_4$ (0.6 mL; 1 M) was added and stirred for 30 min. The NP suspension was then dialyzed (Spectra/Por® 1 regenerated cellulose dialysis membrane, MWCO 6000-8000 Da, Spectrum) for 24 h, in the dark, against an aqueous solution of mannitol (5 %, w/v), lyophilized for 1 day and stored at 4 ºC before use.

Light-activatable NPs were prepared by adding a PEI-DMNC solution (66.7 μL, 150 mg/mL, in DMSO) to an aqueous solution of DS (5 mL, 0.4 mg/mL) and stirred for 5 min. Then, an aqueous solution of $ZnSO_4$ (120 μL, 1 M) was added and stirred for 30 min. The NP suspension was then dialyzed (Spectra/Por® 1 Regenerated Cellulose dialysis membrane, MWCO 6000-8000

Da, Spectrum) for 24 h, in the dark, against an aqueous solution of mannitol (5 %, w/v), lyophilized for 1 day and stored at 4 ºC before use. In some cases, PEI-DMNC was labelled with Qdot525. For that purpose, an aqueous solution of 1-ethyl-3-(3-dimethyllaminopropyl)carbodiimide hydrochloride (EDC; 500 μL of EDC (10 mg/mL, aqueous solution at pH 6.0)) was added to a suspension of Qdots525 (0.16 mmoles, in 310 μL of PBS). After 5 min, PEI-DMNC solution (200 μL, 25 mg/mL in DMSO) was added to the previous solution and allowed to react for 1 h, in the absence of light, at room temperature.

For the preparation of RA-containing NPs ($RA^{+}NPs$), a RA solution (24 μL, 50 mg/mL, in DMSO) was added to a solution of PEI-DMNC (66.7 μL, 150 mg/mL in DMSO) and maintained at room temperature for 30 min, under stirring. The solution was then carefully added to an aqueous solution of DS (5 mL, 0.4 mg/mL) and stirred for 5 min. The NPs in suspension were treated with an aqueous solution of $ZnSO_4$ (120 μL; 1 M) for 30 min. RA that was not encapsulated in the NPs was removed by centrifugation (12,000 g for 3 min). The NP suspension was then dialyzed (Spectra/Por® 1 Regenerated Cellulose dialysis membrane, MWCO 6000-8000 Da, Spectrum) for 24 h, in the dark, against an aqueous solution of mannitol (5%, w/v), lyophilized for 1 day and stored at 4 ºC before use.

For the preparation of fluorescently labelled NPs, NPs (2 mg) were resuspended in 0.1 M carbonate/bicarbonate buffer (1 mL, pH 8.3) followed by the addition of FITC or TRITC (5 μL in DMSO, 3-fold molar excess). The NP suspension was stirred for 1 h in the absence of light and then dialyzed (Spectra/Por® 1 regenerated cellulose dialysis membrane, MWCO 6000-8000 Da, Spectrum) for 24 h against an aqueous solution of mannitol (5 %, w/v), lyophilized, and stored at 4 ºC before use.

Characterization of the NPs. The diameter of the NPs was measured by photon correlation spectroscopy (PCS) using quasi-elastic light scattering equipment (Zeta-Pals™ Zeta Potential

Analyzer, Brookhaven Instruments Corp., Holtsville, NY) and ZetaPlus™ Particle Sizing Software (version 4.03). To measure NP diameter, the NP suspension (2 mL, 50 μg/mL in water for molecular biology) was added to a cuvette and allowed to stabilize for 10 min. The sample was then vortexed for 5 s and subjected to NP size analysis in the ZetaPlus™ for 3 min (3 times; all data were recorded at 90º). After each reading the cuvette was again vortexed for 5 s and exposed to UV light (365 nm) or blue light (405 nm) for a certain period of time (see above). The values of NP diameter and NP counts were recorded. The average diameters described in this work are number-weighted average diameters. The zeta potential of NPs was determined in a 1 mM KCl pH 6 solution, at 25 °C (2 mL, 50 μg/mL). All data were recorded with at least 5 runs (in triplicate) with a relative residual value (measure of data fit quality) of 0.03. The diameter of NPs was also confirmed on a FEI-Tecnai G2 Spirit Bio Twin at 100 kV. NP suspensions (in H_2O) were placed on a 200-mesh copper grid coated with a carbon support film (Taab Labs Ltd.) and dried overnight.

Release of RA from RA^+NPs. $[^3H]$RA solution in DMSO was used for the preparation of NPs, using a 1:20 ratio of labelled to unlabelled RA (1 nCi/μg RA). The initial RA cargo in the NPs was quantified using 2/3 of the original NP suspension (1 mg/ml). To quantify the controlled release of the RA, a suspension of $[^3H]$RA-NPs (10 μg/mL) was prepared and irradiated with UV light (365 nm) or blue light (405 nm). For each time point (0s, 30s, 60s, 180s, 300s, 600s) the NP suspension was centrifuged at 14.000g for 3 min, the supernatant collected and mixed with liquid scintillation fluid (1 mL; Packard Ultima Gold) and the scintillations counted in a TriCarb 2900 TR Scintillation analyser (Perkin Elmer).

Cell culture. HUVECs (Lonza) were cultured in EGM-2 medium (Lonza) in a CO_2 incubator at 37 ºC, 5% CO_2 in a humidified atmosphere, with media changes performed every other day. Cells were passaged every 2-5 days and used for experiments between passage 4 and 6. Human bone marrow acute promyelocytic leukemia NB4 cells, kindly provided by Dr. Arthur Zelent (Institute of Cancer

Research, Royal Cancer Hospital), were cultured in RPMI-1640 (Gibco) supplemented with 10 % fetal bovine serum (Gibco) and 100 U/mL PenStrep (Lonza). Human myelomonoblastic cell lines U937-MT and U937-B412, kindly provided by Dr. Estelle Duprez (Centre de Recherche en Cancérologie de Marseille, France), were maintained at exponential growth in RPMI-1640 medium supplemented with 10% fetal bovine serum and 100 U/mL of PenStrep. For PLZF/RARA induction cells were stimulated with 0.1 mM $ZnSO_4$ for at least 24 h. Human acute monocytic leukemia cell line THP-1 (DSMZ no. ACC16) was cultured in RPM1-1640 media supplemented with HEPES (10 mM), sodium pyruvate (1.0 mM) and 2-mercaptoethanol (0.05 mM).

NP cytotoxicity studies. NPs were suspended in a solution of milli-Q water with PenStrep (5 μL/mL of 10000 U/mL stock solution, Lonza) and Fungizone (2.5 μg/mL, Sigma-Aldrich) for 30 min, centrifuged (14,000 g for 10 min), and finally resuspended in serum free cell culture medium. K562, NB4 and U937 cells ($0.1 \cdot 10^6$ cells/condition) were incubated in serum free RPMI-1640 for 4 h in a 96-well plate containing variable amounts of PEI-DMNC:DS NPs. Once the incubations were terminated the cells were washed gently with medium to remove NP excess, and half of the samples were exposed to UV light (365 nm, 100 Watts) for 10 min. The cells were then cultured for 20 h in 100 μL of complete medium (RPMI-1640 medium supplemented with 10 % fetal bovine serum and 100 U/mL PenStrep). A CellTiter-Glo® luminescent cell viability assay (ATP, Promega, USA) was performed according to the recommendations of the vendor.

Assessment of histone γH2AX phosporylation (DNA damage) induced by UV light or blue light irradiation. HUVEC cells (passage 4) were cultured on 1% gelatin-coated slides until subconfluency in EGM-2, followed by exposure to UV light (365 nm, 100 Watts) or blue light (405 nm, 80 mW) up to 60 min. Control conditions did not receive any light radiation. Following treatment, the medium was replaced by fresh medium and the cells were incubated for additional 6

h on normal culture conditions. The cells were then fixed with 4% paraformaldehyde (Electron Microscopy Sciences) for 10 min at room temperature and then washed with PBS. The cells were then permeabilized with 1 % (v/v) Triton-X, blocked with PBS + 2 % BSA and stained for 1 h with anti-human primary γH2AX antibody (clone: N1-431, BD Biosciences). Detection was done with secondary antibody anti-mouse Cy3 conjugate (Jackson ImmunoResearch). Cell nuclei were stained with 4',6-diamidino-2-phenylindole (DAPI) (Sigma), and the slides were mounted with mounting medium (Dako) and examined with a Zeiss inverted fluorescence microscope.

NP internalization studies. NP internalization was monitored by inductive coupled plasma mass spectrometry (ICP-MS). In this case, the intracellular levels of Zn were measured before and after cell exposure to NPs. NB4 and U937 cells ($0.1 \cdot 10^6$ cells/well) were plated in 24 well plates and incubated in serum free RPMI-1640 from 1 to 24 h with variable amounts of PEI-DMNC:DS NPs. After incubations, NPs that were not internalized by the cells were washed (three times with PBS) and the cells were centrifuged; followed by the addition of an aqueous solution of nitric acid (1 mL, 69 % (v/v)). The samples were analyzed by ICP-MS for the concentration of intracellular levels of Zn. The concentration of Zn was normalized per cell. The estimation of NPs was done based on controlled standard solutions.

Mechanism of NP uptake. U937 cells were cultured on 24 well plates ($1 \cdot 10^5$ cells/well) and inhibited by one of the following chemicals during 30 min before adding a suspension of TRITC-labelled NPs (5 μg/mL): EIPA (50 μM), dynasor (80 μM), dansylcadaverine (100 μM), cytochalasin D (10 μM), nocodazole (50 uM), filipin III (100 μM) and polyinosinic acid (100 μg/mL). The inhibitor concentrations were based in values reported in literature and further validated by us to have no cytotoxic effect over the period of the assay (6 h), as confirmed by ATP assay. The incubation of the cells with NPs for different times was performed in the presence of the inhibitor.

As controls, we used cells without NPs and cells incubated with NPs without inhibitor. At the end of each time point, cells were centrifuged at 1300 rpm, 20 ºC for 5 min with PBS, washed one time with cold trypan blue solution (200 μL; 600 μg/mL), re-washed 3 times with cold PBS and then resuspended in PBS containing 2.5 % FBS (500 μL) for flow cytometry analysis. A total of 10,000 events were obtained per measurement. To validate the inhibitory activity of dynasor we performed uptake studies of FITC-labelled transferrin, known to selectively enter cells via clatherin-mediated endocytosis. Briefly, U937 cells were cultured on 24 well plates ($1 \cdot 10^5$ cells/well) and treated or not with dynasor (80 μM, 30 min pre-incubation), followed by addition of 1 μg/mL FITC-labelled transferrin (Life Technologies). The transferrin was allowed to bind for 3 min at 4 ºC. Cells were then evaluated as before.

The NP uptake mechanism was also studied on U937 cells by silencing specific proteins of clathrin-mediated endocytosis (CLTC and LDLR), caveolin-mediated endocytosis (CAV1), GEEC-CCLIC pathways (CDC42) and macropinocytosis (RAC1 and CTBP1) by siRNA (Thermo Fisher). Transfection was performed in a 24 well plate with $0.5 \cdot 10^5$ cells in antibiotic-free complete medium with 100 nM siRNA and 1.5 μL of Lipofectamine RNAiMAX (Life Technologies) transfection reagent for 24 h. After this initial period, the transfection medium was replaced by complete medium and the cells incubated for another 48 h. Then, cells were cultured with TRITC-labelled NPs (5 μg/mL) for 6 h. Once the incubations were terminated, the cells were centrifuged at 1300 rpm, 20 ºC for 5 min, with PBS, washed one time with cold trypan blue solution (200 μL; 600 μg/mL), re-washed 3 times with cold PBS and then resuspended in PBS containing 2.5 % FBS (500 μL) for flow cytometry analysis. Non-transfected cells or cells transfected with lipofectamine but without siRNAs (MOCK) were used as controls. In all flow cytometry analysis, a total of 10,000 events were recorded per run. All conditions were performed in triplicate.

Intracellular trafficking analyses of NPs. HUVEC cells (passage 4) were cultured on 1 % gelatin-

coated slides until subconfluency in EGM-2. The cells were then incubated with FITC-labelled NPs (1 μg/mL) for 1 or 4 h, washed extensively, exposed or not to UV light (365 nm, 100 Watts) and cultured in normal conditions for up to 12 h. For LysoTracker staining, at time points 2, 6 and 12 h, the cells were incubated with LysoTracker Red DND-99 (50 nM, Invitrogen). After 30 min of incubation, the coverslips were washed extensively with PBS, followed by cell fixation with paraformaldehyde (4%, Electron Microscopy Sciences) for 10 min at room temperature and then washed with PBS. Cell nuclei were stained with 4',6-diamidino-2-phenylindole (DAPI) (Sigma), and the slides were mounted with mounting medium (Dako) and examined with a Zeiss LSM 50 confocal microscope.

Co-localization analysis was performed by culturing HUVEC cells (passage 4) on 1 % gelatin-coated slides until subconfluency in EGM-2. Cells were treated with 1 μg/mL FITC-labeled NPs for 1 or 4 hours, washed extensively and cultured in normal conditions for 1 or 1/8 additional hour/s, respectively. Then the cells were fixed with 4 % paraformaldehyde (Electron Microscopy Sciences) for 10 min at room temperature, blocked with 2 % (w/v) BSA, and when necessary, permeabilized with 0.5 % (v/v) Triton-X. Cells were then stained for 1 h with anti-human primary antibodies against EEA1 (clone: C45B10, Cell Signaling), rabankyrin-5 (Rab 5, ANKFY1 (D-15), Santa Cruz Biotechnology), or rabankyrin-7 (Rab 7, clone: D95F2, Cell Signaling). In each immunofluorescence experiment, an isotype-matched IgG control was used. Binding of primary antibodies to specific cells was detected with anti-rabbit or anti-goat IgG Cy3 conjugate (Jackson ImmunoResearch). Cell nuclei were stained with 4',6-diamidino-2-phenylindole (DAPI) (Sigma), and the slides were mounted with mounting medium (Dako) and examined with a Zeiss LSM 50 confocal microscope. Co-localization analysis was done in ImageJ through assessment of the percentage of overlapping objects. Two objects are considered to be co-localizing when their intensity profile is overlaping more than 40 %. For this analysis the number (percentage of FITC-labeled NPs foci that are positive for EEA-1/Rab-5/Rab-7) and the intensity volume (percentage of

FITC-labeled NPs in the EEA-1/Rab-5/Rab-7-positive compartments) were used. This approach was found to be more adequate than classical co-localization tools in ImajeJ or other softwares that measure pixel co-occurrence and correlation analyses, because it allowed us to (i) discriminate between background and vesicle/NP-foci fluorescence and (ii) interpret the results in terms of percentage of NP-foci that are localized to vesicles in another channel of interest.

NP dilution during cell proliferation. NP dilution with cell growth was monitored over 6 days by inductive coupled plasma mass spectrometry (ICP-MS) by the quantification of intracellular levels of Zn. NB4 and THP-1 cells (0.5×10^6 cells/mL) were plated in 6 well plates and incubated in serum free RPMI-1640 with 20 μg/mL of RA^+NPs. After 4 h incubation, NPs that were not internalized by the cells were washed three times with PBS and the cells were left to grow at 0.2×10^6 cells/mL in complete medium for additional 4 h, 3 days and 6 days, maintaining always an exponential growth. After each incubation, cells were counted, collected by centrifugation and ressuspended in nitric acid (1 mL, 69 % (v/v) for ICP analysis. The concentration of Zn was normalized per cell. The estimation of NPs was done based on Zn quantification in 20 μg of NPs. In some experiments, cells were transfected with RA^+NPs labeled with TRITC, and their fluorescence monitored by flow cytometry overtime, to evaluate NPs distribution within the cells.

Exocytosis analyses of NPs. To determine exocytose of NPs, NP uptake assays were performed in the presence of Pgp antagonist verapamil or the endosome disruption agent chloroquine. U937 cells were cultured on 24 well plates ($1 \cdot 10^5$ cells/well) and chloroquine (100 μM, no pre-incubation) and verapamil (100 μM, 60 min pre-incubation) conditions were tested. The chemical agents concentrations were based on values reported in the literature and further validated by us to have no cytotoxic effect over the period of the assay (12 h). After the pre-incubation with the chemical agents, TRITC-labelled PEI-DMNC:DS NPs (10 μg/mL) or TRITC-labelled poly-L-lysine USPIO

NPs (100 μg/mL) were added to the cells, maintaining the chemical agents concentration. As controls we used cells incubated without NPs and cells incubated with NPs without chemical agents. At the end of each experiment, the cells were centrifuged at 1300 rpm, 20 ºC for 5 min with PBS, washed one time with cold trypan blue solution (200 μL; 600 μg/mL), re-washed 3 times with cold PBS and then resuspended in PBS containing 2.5 % FBS (500 μL) for flow cytometry analysis. A total of 10,000 events were recorded per measurement, and all conditions were performed in triplicate.

[^{3}H]RA internalization studies. [11, 12-^{3}H(N)]-Retinoic acid, 50.4 Ci/mmol, was purchased from Perkin Elmer. [^{3}H]RA solution for cell culture assays was prepared on the day of experiments by dissolving [^{3}H]RA in DMSO with unlabelled RA in a 1:1000 ratio to a final concentration of 10 μM of RA. [^{3}H]RA solution in DMSO for the preparation of NPs was prepared on the day of experiments using a 1:4000 ratio of labelled to unlabelled RA. Experiments were initiated by the adding the [^{3}H]RA solution (1 μM and 10 μM; representing less than 1% in volume of the total cell culture medium) or [^{3}H]RA-NP suspension (1 μg/mL and 10 μg/mL) to cultures (60,000 cells/condition, 24-well plate, 1 mL) of NB4 or U937 cells. In case of soluble RA, cells (NB4 or U937; 60,000 cells/condition, 24-well plate) were cultured with medium containing [^{3}H]RA (1 μM and 10 μM; 1 mL of medium) for 24 or 72 h, washed with PBS (2 times), harvested, lysed with lysis buffer (100 μL) and kept on ice until scintillation counting procedure. In case of RA-containing NPs, cells (same conditions as for soluble RA) were cultured with [^{3}H]RA-NPs (1 μg/mL and 10 μg/mL) for 4 h, washed with PBS and cultured for additional 20 or 68 h in the respective culture medium. Cells were then collected to eppendorfs, washed with PBS, centrifuged (1500 rpm, 5 min), lysed with lysis buffer (see above) and kept on ice until scintillation counting procedure. The lysed samples (100 μL) were mixed with liquid scintillation fluid (1 mL; Packard

Ultima Gold) and the scintillations counted in a TriCarb 2900 TR Scintillation analyser (Perkin Elmer).

Time-activation of NPs within cells. NB4 and Zn-induced U937-B412 cells ($6.0 \cdot 10^4$ cells/condition) were plated in 24-well plates and transfected with RA^+ NPs (1 μg/mL) for different time periods (1, 2, 4, 6, 8, 12 and 24 h). The cells were then washed by centrifugation (1300 rpm, 5 min) to remove non-internalized NPs, and immediately exposed to UV light (365 nm, 100 Watts, 5 min). In a second experimental setup, NB4 and Zn-induced U937-B412 cells ($6.0 \cdot 10^4$ cells/condition) were plated in 24-well plates and transfected with RA^+ NPs (1 μg/mL) for 4 h. The cells were then washed by centrifugation (1300 rpm, 5 min) to remove non-internalized NPs, cultured in normal conditions and exposed to UV light (365 nm, 100 Watts, 5 min) at different time points (0, 4, 8, 20 and 44 h). The effect of the intracellular release of RA was evaluated in terms of differentiation of the cells into the myeloid lineage (as assessed by the expression of CD11b) at day 3, as assessed by flow cytometry. All conditions were performed in triplicate.

NB4-RARE cells ($2.5 \cdot 10^4$ cells/condition) were plated in v-shaped 96-well plates and transfected with RA^+ NPs (1 μg/mL) for different time points (1, 2, 4, 6, 8, 12 and 24 h). The cells were then washed by centrifugation (1300 rpm, 5 min) to remove non-internalized NPs, and immediately exposed to UV light (365 nm, 100 Watts, 5 min). For the second experimental setup, NB4-RARE cells ($2.5 \cdot 10^4$ cells/condition) were plated in v-shaped 96-well plates and transfected with RA^+ NPs (1 μg/mL) for 4 h. The cells were then washed by centrifugation (1300 rpm, 5 min) to remove non-internalized NPs, cultured in normal conditions and exposed to UV light (365 nm, 100 Watts, 5 min) at different time points (0, 4, 8, 20 and 44 h). The cells were then cultured for 12 hours after each condition light activation in RPMI-1640 medium supplemented with 10 % fetal bovine serum and 100 U/mL PenStrep. After these procedures luciferase luminescence was quantified as described above for the luciferase assays. All conditions were performed in triplicate.

Multiple activation of NPs within cells. Myelocytic differentiation of Zn-induced U937 cells was assessed by the quantification of CD11b expression by flow cytometry. U937-B412 cells ($6.0 \cdot 10^4$ cells/condition) were cultured with $ZnSO_4$ (0.1 mM) in culture medium up to 24 h prior to experiment to induce the expression of promyelocytic leukemia zinc finger/RARα (PLZF/RARα). Then cells were transfected with RA^+ NPs (1 μg/mL) for 4 h, washed, placed in normal culture medium and then different activated by UV light (365 nm, 100 Watts, 5 min). Cells without light activation were used as control. The following conditions were tested: i) single light activation at 4 h; ii) light activations at 4 h and 6 h; iii) light activations at 4 h, 6 h and 8 h and iv) light activations at 4 h, 6 h, 8 h and 10 h. After 3 days, expression of CD11b on U937 cell surface was measured by staining with a fluorescent (PE)-conjugated anti-CD11b mAb (BD Biosciences) using flow cytometry. All conditions were performed in triplicate.

NB4 differentiation assay. Myelocytic differentiation of NB4 cells was assessed by quantifying $CD11b^+$, $CD11b^+CD45^+CD13^{high}$ or $CD11b^+CD45^+CD13^{low}$ populations using flow cytometry. NB4 cells (between $6.0 \cdot 10^4$ and $10 \cdot 10^4$ cells/condition) were plated in 24-well plates and cultured with soluble RA (3 μg/mL) or light-activatable RA^+ NPs (10 μg/mL) for 3 days. The NPs were suspended in serum free medium and added to cells for 4 h. The cells were then washed by centrifugation (1300 rpm, 5 min) to remove non-internalized NPs, and half of the samples were exposed to UV light (365 nm, 100 Watts, 5 min). The cells were then cultured up to 3 days in RPMI-1640 medium supplemented with 10 % fetal bovine serum and 100 U/mL PenStrep with half medium changes every 3 days.

NB4 RARE cell line generation. The cignal lenti RARE reporter kit (CLS-016L SABiosciences) was used for the establishment of a RA reporter NB4 cell line. For that purpose, rectronectin

solution (15 μg/cm^2, 30 μg, 500 μL on PBS, Takara) was plated in a 24-well plate 2 hours prior to cell seeding. The plate was kept at room temperature and was washed one time, immediately before seeding, with PBS. NB4 cells (1 · 10^5) were plated in 175 μL of RPMI-1640 medium (Gibco) supplemented with 0.5 % FBS and 100 U/mL PenStrep and to this condition 125 μL of cignal lentiviral particles were added to a total experimental volume of 300 μL. After a gentle swirl of the plate the cells were incubated 20 hours at 37 ºC in a humidified incubator with 5 % CO_2 atmosphere. In the following day, cells were washed and allowed to recover in the incubator for 24 hours cultured in 500 μL of fresh RPMI-1640 medium supplemented with 10 % FBS and 100 U/mL PenStrep. After that, 2 μg/mL of puromycin (Invitrogen) was added to the culture medium for selection of transduced cells. Evaluation of selection efficiency in puromycin-containing medium was performed every 3 days for a period of 5 weeks.

NB4 RARE luciferase assay. To assess the biological effect of RA in RAR-regulated signalling pathway activity, luciferase reporter assay was performed. NB4-RARE cells (2.5 · 10^4 cells/condition) were plated in v-shaped 96-well plates and cultured with soluble RA (10 μM) or light-activatable RA$^+$ NPs (5 μg/mL). The NPs were suspended in serum free medium and added to cells for 1 h. The cells were then washed by centrifugation (1300 rpm, 5 min) to remove non-internalized NPs, and half of the samples were exposed to blue light (405 nm, 80 mW, 5 min). The cells were then cultured for 12/24 hours in RPMI-1640 medium supplemented with 10 % fetal bovine serum and 100 U/mL PenStrep. After these incubation times, the conditions were centrifuged (1500 rpm, 3 min), excess medium carefully aspirated and the cells washed with 100 μL of PBS. After a new centrifugation and removal of PBS, 60 μL of cell lysis buffer (8 mM of magnesium chloride; 1 mM DL-Dithiothreitol; 1 mM Ethylenediaminetetraacetic acid; 25 mM of 1 M Trizma Base with 1 M Sodium phosphate monobasic; 15 % Glycerol; and 1 % Triton X-100), was added to each condition. The plate was kept on ice, under agitation for 15 min to allow

complete lysis and then the plate was placed on -80 ºC for the amount of time necessary for the samples to freeze. After these steps, the plate was removed from the -80 ºC, put on ice and allowed to defrost at slow rate.

For the preparation of the luminescence reading, 40 µL of ATP (100 µM, Sigma) was added to 1960 µL of reading buffer solution (8 mM of magnesium chloride; 1 mM DL-Dithiothreitol; 1 mM Ethylenediaminetetraacetic acid; 25 mM of 1 M Trizma Base with 1 M Sodium phosphate monobasic; and 15 % Glycerol) to a final concentration of 2 µM ATP. On a second tube, 2 mL of D-Luciferin working solution (167 µM, Sigma) was prepared protected from light. The injection system of the luminometer was primed until ready. Following that step, the luminometer software was programmed to set the temperature to 37 ºC, and under stirring for the duration of the experiment accept 50 µL of sample per condition in a 96-white plate, inject 100 µL of ATP working solution 3 seconds after reading cycle begins; inject 100 µL of D-Luciferin working solution 4 seconds after reading cycle begins and read the luminescence 5 seconds after reading cycle begins. The luciferase luminescence was quantified in a microplate luminometer reader LumiStar Galaxy (BMG Labtech). All conditions were performed in triplicate.

NB4 RARE differentiation by cell conditioned medium obtained from NB4 cells exposed to RA or RA^{+}NPs. NB4 cells ($3 \cdot 10^6$/mL) were grown in RPMI supplemented with 10% (v/v) FBS, penicillin and streptomycin. Cells were treated with RA^{+}NPs (10 µg/mL) for 4 h, washed with PBS to remove the NPs that were not internalized, activated by a blue laser for 5 min, and then cultured for 18 h under hypoxia conditions (0.5% O_2) in exosome-depleted medium. A separate group of cells was treated with RA (3 µg/mL) for 22 h in hypoxia conditions (0.5% O_2) in exosome-depleted medium. Then, in both groups, cells were centrifuged at 10.000 g (4ºC; 30 min) and part of the supernatant (named in the plot as "Sup") added to NB4 RARE cells (100,000/mL; 1 mL of the Sup). Alternatively, in both groups, cells were centrifuged in multiple steps[3] to remove exosomes (named

in the plot as "exo") and added to NB4 RARE cells (100,000/mL; 5 μg/mL de exosomes per mL of media) plated in a 24 well plate. After 24 h, the luciferase assay was performed according to instructions of the luciferase kit (britelite™).

U937 differentiation assay. Myelocytic differentiation of U937 cells was assessed by the quantification of CD11b expression by flow cytometry. U937-B412 cells ($6.0 \cdot 10^4$ cells/condition) were cultured either with or without $ZnSO_4$ (0.1 mM). To induce the expression of promyelocytic leukemia zinc finger/RARα (PLZF/RARα) in U937-B412 cells they were treated for 24 h with $ZnSO_4$ (0.1 mM). Then cells were treated with soluble RA or light-activatable RA^+ NPs (transfection for 4 h followed by light activation for 5 min) for 3 days. After 1 and 3 days, expression of CD11b on U937 cell surface was measured by staining with a fluorescent (PE)-conjugated anti-CD11b mAb (BD Biosciences) using flow cytometry. All conditions were performed in triplicate.

AML differentiation assay. AML bone marrow mononuclear cells isolated by Ficoll-Histopaque (GE Healthcare) gradient centrifugation, enriched using the MACS CD34 isolation kit (Miltenyi Biotec) and cryopreserved were kindly provided by Dr. Rajeev Gupta (Department of Haematology, UCL Cancer Institute). The AML cells were isolated from a 85 years old man patient with AML 34+117+33+13+DR+ 35% blasts and a 70 years old woman with RAEB2/evolving AML 34+117+33+ 12% blasts. Both samples had formal karyotyping/extended FISH panels. Briefly, neither patients were PML-RARA: one was standard risk (normal karyotype with a small trisomy 8 subclone), and the other was high risk with complex karyotype. The isolated $CD34^+$ AML cells were maintained in StemSpan SFEM medium (Stemcell Technologies) supplemented with a human cytokine cocktail containing SCF (50 ng/mL, Stemcell Technologies), TPO (15 ng/mL) and Flt-3L (50 ng/mL, PeproTech) plus PenStrep (10,000 U/mL, Lonza) and Fungizone (25 μg/mL,

Sigma) up to 3 days. Prior to the colony-forming cell (CFC) and long-term culture-initiating cell (LTC-IC) assays, AML cells were incubated for 4 h in Ex-Vivo (Lonza) serum free medium, with and without blank NPs or RA^+ NPs in a 24 well plate. After that time, the cells were washed to remove loosely bound NPs. For CFC assays (2.0 x 10^5 cells/condition) AML cells were plated in triplicate in MethoCult H4230 medium (3 mL, StemCell Technologies) supplemented with SCF [50 ng/mL], IL-3 [10 ng/mL], and Flt-3L [50 ng/mL], all human, plus PenStrep (10,000 U/mL, Lonza) and Fungizone (25 μg/mL, Sigma) in 6-well plate. In some conditions, RA^+ NPs accumulated within the cells were activated by a UV light (365 nm, 100 W, 5 min). Cultures were scored after 14 days for the presence of clusters and colonies containing >20 cells using an inverted microscope. LTC-IC assays were performed in triplicate in a 6-well plate gelatinized for 2 h prior to adding the feeders. The feeder layer was composed of a 1:1 mixture of irradiated (80 Gy) SL/SL ($1.5 \cdot 10^4$ cells/condition) and M210B4 mouse fibroblasts ($1.5 \cdot 10^4$ cells/condition), kindly provided by Dr. Rajeev Gupta (Department of Haematology, UCL Cancer Institute). AML cells ($1 \cdot 10^6$ cells/condition) were plated in Myelocult H5100 medium (StemCell Technologies), supplemented with Flt-3L [50 ng/mL], hydrocortisone [10^{-6} M] (StemCell Technologies) and PenStrep (10,000 U/mL, Lonza) and fungizone (25 μg/mL, Sigma). For some conditions UV light (365 nm, 100 Watts, 5 min) was used to trigger RA release. After the cells were inoculated, weekly half medium changes were performed (with Flt-3L [100 ng/mL]) for the duration of the culture. After 5 weeks, all cells were harvested and placed into methylcellulose based assay for the detection of AML-CFC as described above.

High-throughput gene expression assay. Gene expression was evaluated by real time PCR in NB4 cells and U937-B412 cells previously stimulated with $ZnSO_4$ (0.1 M), using a 96.96 microfluidic chip (Fluidigm Corporation). Cells were exposed to either soluble RA (1 and 10 μM) or light-activatable RA^+NPs (0.1, 1 and 10 μg/mL) for 8 h, 24 h or 48 h. From each sample (*n*=3) a

maximum of 0.45 × 10^6 cells were collected for RNA extraction using RNeasy Micro Kit. Afterwards, samples were processed using Fluidigm standard protocols. Briefly, cDNA was obtained from 50 ng of RNA using Reverse Transcription Master Mix (Fluidigm Corporation). Then samples were pre-amplified for 12 cycles, to increase the number of copies of target DNA, with Fluidigm PreAmp Master Mix (Fluidigm Corporation) and a pool of all the primers tested in the chip. Prior to qPCR reactions the pre-amplification reaction was treated with Exonuclease I to eliminate the carryover of unincorporated primers. Finally, samples and genes pre-mix are prepared separately accordingly to manufactures instructions using SsoFast EvaGreen Supermix with Low ROX (Bio-Rad Laboratories) and loaded into the chip. A pneumatically operated desktop instrument (IFC Controller HX) was used to mix samples and genes pre-mix in the qPCR chamber reaction of the chip. After this procedure, the chip was analyzed using Biomark HD (Fluidigm Corporation) for the thermal cycling and real time fluorescent readings. A melting curve was performed after 30 cycles, for quality control. DeltaCt was calculated using Real Time PCR Analysis Software 4.1.3 (Fluidigm Corporation) and data was further analyzed with Cluster 3.0 and Java Trew View to produced gene expression heatmaps and hierarchical clustering.

Generation of a THP-1- GFP cell line. Viral particles for GFP-expression were produced in 293T cells using a standard protocol. Briefly, 293T cells were transfected with GFP DNA construct (30 μg) and pCL-Eco (DNA:pCL-Eco; 15 μg) both solubilised in a DNA-$CaCl_2$ mixture (1 mL). Transfection efficiency was evaluated by fluorescence microscopy. After 72 hours viral particles were collected and THP-1 cells were infected (2 x 10^5 cells/ml in a 6-well plate with 4μg/ml polybrene).

***In vivo* study: subcutaneous implantation.** The animal work has been conducted according to relevant national and international guidelines and approved by the Bioethics Committee of

University of Salamanca. On the day before injecting the cells, PDMS cylindrical constructs (∅internal = 1.0 cm; ∅external = 1.5 cm) were implanted subcutaneously on NOD/SCID mice (Jackson Laboratory) maintained in pathogen-free conditions with irradiated chow. For the *ex-vivo* activation studies in the day of the experiment, NB4 cells were suspended in serum free medium with (i) no NPs, (ii) with empty NPs (10 µg/mL) or RA^+ NPs (10 µg/mL) for 4 h. At the end, cells were washed by centrifugation (1300 rpm, 5 min), and the ones treated with RA^+ NPs were either activated or not with a blue laser (405 nm, 80 mW) for 5 min. NB4 cells ($5 \cdot 10^6$ cells per PDMS construct) were injected subcutaneously in the center of the PDMS construct embedded in Matrigel (200 µL, BD Biosciences). Five days after injection of the cells, animals were sacrificed by cervical dislocation and cells within the cylindrical construct were collected and characterized by flow cytometry. For the *in vivo* activation studies in the day of the experiment, NB4 cells were suspended in serum free medium with (i) no NPs, (ii) with RA^+ NPs (10 µg/mL) for 4 h. At the end, cells were washed by centrifugation (1300 rpm, 5 min), and $5 \cdot 10^6$ NB4 cells per PDMS construct were injected subcutaneously in the center of the PDMS construct embedded in Matrigel (200 µL). One day after injection, experimental groups were either activated or not with a blue optical fiber (405 nm, 80 mW) for 5 min. Three days after injection of the cells, animals were sacrificed by cervical dislocation and cells within the cylindrical construct were collected and characterized by flow cytometry.

***In vivo* study: bone marrow modulation.** NOD.CB17-Prkdcscid /J (NOD/SCID) female mice (*n*=12) aged 6-8 weeks were employed in this experiment. Before cell injection, mice received 1.5 Gy of total body irradiation from a 137Cs source and were also treated with 200 µg of mouse anti-CD122 monoclonal antibody (NS122) by intraperitoneal injection. Human THP-1 cells were incubated with 20 µg/mL of RA^+NPs in RPMI medium for 4 hours, followed by extensive wash with PBS to remove non-internalised NPs. The cells were then resuspended in RPMI medium with

10% FBS and left in the culture incubator overnight. On the following day, the cells loaded with RA^+NPs were collected and $1 \cdot 10^7$ cells /mouse in 200 μL PBS were injected into the NOD/SCID mice intravenously, through the tail vein. After 6 days, the mice were randomly divided into two groups: one of the groups was blue laser irradiated (*n*=6) and the other group was not irradiated (n=6). In the blue laser irradiated group, each mouse was anesthetized with 3.5% chloral hydrate in PBS and the craniums were exposed to a blue laser (405 nm, 80 mWatts) for 5 min. The mice in the non-irradiated group received the same treatment without blue laser activation. After 48 or 72 h, the mice were sacrificed and the long bones/ craniums were collected. The long bones including the femurs, tibias and pelvis were crushed in PBS containing 1% bovine serum albumin (BSA, sigma) and 2 mM EDTA (Invitrogen). The samples were then filtered with 70 μm cell strainer and treated with ACK solution to lyse red blood cells. The resulting cells were stained using PE conjugated anti-human CD45 antibody (eBioscience). The results were measured by flow cytometry and the data were analyzed with FlowJo software.

The mouse craniums were collected for the *ex vivo* staining examination followed by the traditional protocol. Briefly, the mouse craniums were cut into four pieces along the sutures producing one frontal, two parietal and one occipital bone. Only parietal bones were used in this experiment. Bone pieces were fixed in 4% paraformadehyde for 30 min, washed twice with PBS, blocked with 2% BSA-0.01% Triton X-100 in PBS (BSA buffer) for 1 hr, and incubated with primary antibodies (diluted 1:100 in BSA buffer) overnight at 4ºC. After washing with BSA buffer for 2 hr, bones were incubated with secondary antibodies (diluted 1:200 in BSA buffer) for 2 hr, washed with BSA buffer for 2 hr, and nuclei were stained using DAPI. The primary antibodies included mouse anti-human CD45 antibody (BD) and rabbit anti- human CD11b antibody (Abcam). The secondary antibodies included goat anti-mouse 555 (invitrogen) and goat anti-rabbit 488 antibodies. The bone pieces were finally examined with a confocal microscope (Nikon Eclipse Ti) under FITC/TRITC/DIC signal channel. Images were analysed in imageJ. Analyse Particles was

used after thresholding to quantify positive signals and measure the total area occupied by these particles.

***In vivo* study: CRABP2 expression on mouse cranium.** NOD.CB17-Prkdcscid /J (NOD/SCID) female mice aged 6-8 weeks were employed in this experiment. The mouse craniums were collected from the last experiment. In this experiment, we also collected the mouse craniums without any treatment as the control. The ex vivo staining examination was followed by the traditional protocol. Briefly, the mouse craniums were cut into four pieces along the sutures producing one frontal, two parietal and one occipital bone. Only parietal bones were used in this experiment. Bone pieces were fixed in 4% paraformadehyde for 30 min, washed twice with PBS, blocked with 2% BSA-0.01% Triton X-100 in PBS (BSA buffer) for 1 hr, and incubated with primary antibodies (anti-CRABP2 antibody, ab74265, diluted 1:100 in BSA buffer) overnight at 4ºC. After washing with BSA buffer, bones were incubated with secondary antibodies (goat anti-rabbit 488 antibodies, diluted 1:200 in BSA buffer) for 2 hr and then washed with BSA buffer. Nuclei were stained using DAPI. The bone pieces were finally examined with a confocal microscope (Nikon Eclipse Ti) under DAPI/FITC/DIC signal channel. Images were analysed in imageJ. Analyse Particles was used after thresholding to quantify positive signals and measure the total area occupied by these particles. Comparison between CD11b, CD45 and CRABP2 positive areas was used as a proxy of the release of RA by NPs and paracrine effect in the parietal bones of irradiated and non-irradiated mice.

SUPPLEMENTARY REFERENCES:

1. Maia, J. et al. Controlling the neuronal differentiation of stem cells by the intracellular delivery of retinoic acid-loaded nanoparticles. *ACS Nano* **5**, 97-106 (2011).
2. Ruthardt, M. et al. Opposite effects of the acute promyelocytic leukemia PML-retinoic acid receptor alpha (RAR alpha) and PLZF-RAR alpha fusion proteins on retinoic acid signalling. *Mol Cell Biol* **17**, 4859-4869 (1997).
3. Thery, C., Amigorena, S., Raposo, G. & Clayton, A. Isolation and characterization of exosomes from cell culture supernatants and biological fluids. *Curr Protoc Cell Biol* **Chapter 3**, Unit 3 22 (2006).
